# Pathology and causes of death in stranded humpback whales (*Megaptera novaeangliae*) from Brazil

**DOI:** 10.1371/journal.pone.0194872

**Published:** 2018-05-16

**Authors:** Kátia Regina Groch, Josué Díaz-Delgado, Milton C. C. Marcondes, Adriana Castaldo Colosio, Elitieri B. Santos-Neto, Vítor Luz Carvalho, Gisele Silva Boos, Ana Carolina Oliveira de Meirelles, Hernani Gomes da Cunha Ramos, Juliana Plácido Guimarães, João Carlos Gomes Borges, Jociery Einhardt Vergara-Parente, Judy A. St. Leger, Antonio Fernández, José Luiz Catão-Dias

**Affiliations:** 1 Laboratory of Wildlife Comparative Pathology, Department of Pathology, School of Veterinary Medicine and Animal Science, University of São Paulo, São Paulo, Brazil; 2 Instituto Baleia Jubarte, Caravelas, Bahia, Brazil; 3 Division of Histology and Animal Pathology, Institute for Animal Health, Veterinary School, University of Las Palmas de Gran Canaria, Arucas, Canary Islands, Spain; 4 Associação de Pesquisa e Preservação de Ecossistemas Aquáticos, Caucaia, Ceará, Brazil; 5 Setor de Patologia Veterinária, Faculdade de Veterinária, Universidade Federal do Rio Grande do Sul, Porto Alegre, Rio Grande do Sul, Brazil; 6 Fundação Mamíferos Aquáticos, Recife, Pernambuco, Brazil; 7 Universidade Santa Cecília, Pós-Graduação em Sustentabilidade de Ecossistemas Costeiros e Marinhos, Santos, São Paulo, Brazil; 8 Instituto de Tecnologia e Pesquisa, Aracaju, Sergipe, Brazil; 9 SeaWorld, San Diego, California, United States of America; Universiteit Gent, BELGIUM

## Abstract

This study describes the pathologic findings of 24 humpback whales (*Megaptera novaeangliae)* found stranded along the Brazilian coast from 2004 to 2016. Eighteen (75%) animals evaluated were found stranded alive. From these, 13 died naturally on shore and five were euthanized. Six died at sea and were washed ashore. Of the 24, 19 (79.2%) were calves, four (16.7%) were juveniles, and one (4.2%) was an adult. The most probable cause of stranding and/or death (CSD) was determined in 23/24 (95.8%) individuals. In calves, CSD included neonatal respiratory distress (13/19; 68.4%), infectious disease (septicemia, omphaloarteritis and urachocystitis; 3/19; 15.8%), trauma of unknown origin (2/19; 10.5%), and vehicular trauma (vessel strike; 1/19; 5.3%). In juveniles and adult individuals, CSD was: emaciation (2/5; 40%), sunlight-thermal burn shock (1/5; 20%); and discospondylitis (1/5; 20%). In one juvenile, the CSD was undetermined (1/5; 20%). This study integrates novel findings and published case reports to delineate the pathology of a South-western Atlantic population of humpback whales. This foundation will aid in the assessment of the population health and establish a baseline for development of conservation policies.

## Introduction

Humpback whales (*Megaptera novaeangliae*) are present in all oceans of the globe and migrate between winter breeding grounds and summer feeding destinations [1]. The South-western Atlantic Ocean population migrates annually from the feeding areas in the Scotia Sea [2,3] to the Brazilian coast with the major concentration occurring in the Abrolhos Bank (16°40’-19°30’S; 37°25’-39°45’W), an enlargement of the continental shelf [4–6]. The whales aggregate in this area to breed and nurse their calves. The population size was estimated at 19,429 whales in 2012 [7]. Strandings have been recorded along the entire Brazilian coast; efforts to gather information on causes of death and comorbidities have increased in the last decade. Nonetheless, causes of mortality and morbidity in this species remain largely unknown.

Most of the published pathology information for the species is limited to cases with prominent externally visible injuries such as those derived from entanglement in fishing gear, vessel strikes and predation by sharks [8–16]. Humpback whales typically strand as single individuals. The only recorded mass stranding event occurred in a feeding area in Cape Cod Bay and Nantucket Sound, US, between November 1987 and January 1988 [17]. During this stranding, 14 whales died after consuming Atlantic mackerel (*Scomber scombrus*) containing saxitoxin, a neurotoxin that blocks the entry of Na^+^ into the nerve and muscle cells [17,18]. The toxin was detected in the kidneys, livers and stomach contents. Histopathology conducted on three animals revealed no significant lesions [17]. Few reports are encountered in the scientific literature regarding the health of Brazilian humpback whales. In 1987, a female calf was reported to have been caught incidentally in a fishing net at Vila Velha, Espírito Santo state [19]. Another specimen, an adult female, stranded in the vicinity of Aracruz, Espírito Santo state, on 01 November 1987, after being harpooned by fishermen [19]. No pathological data were reported in either case. In 2007, a calf was found dead on Itaparica Island, Bahia state, with multiple propeller wounds [20]. In 2014 a young female was found dead in Florianópolis, Santa Catarina state, with evidence of blunt trauma caused by vessel strike [21]. Three humpback whales were reported entangled in fishing gear in the waters off South-eastern Brazil; however, no pathological data were available [22]. Severe discospondylitis was seen in two humpback whales stranded along the Abrolhos Bank seashore [23]. Furthermore, information on health and disease aspects of other cetacean species inhabiting this area is very limited. The first fatal case of cetacean morbillivirus (CeMV) in South America involved a Guiana dolphin (*Sotalia guianensis*) from the coastal waters of Abrolhos Bank [24]. This animal was infected by a new CeMV strain, *Sotalia guianensis*-CeMV. Additionally, evidence of bycatch and use of meat and blubber was observed in two bottlenose-dolphins (*Tursiops truncatus*) found stranded in the Abrolhos Bank seashore [25].

This work focuses on the pathologic findings and causes of death of humpback whales stranded along the Brazilian coastline between 2004 and 2016.

## Materials and methods

This study was approved by the Instituto Chico Mendes de Conservação da Biodiversidade, and conducted under SISBIO licenses #13303–2 and #13303–3. This study is in agreement with Ethical Principles in Animal Research adopted by "Ethic Committee in the use of animals" of the School of Veterinary Medicine and Animal Science of the University of São Paulo (protocol #2212/2011).

Animals stranded between 2004 and 2016, representing the entire Brazilian coast (approximately 7,491 km) and considered suitable for histopathologic analysis, were included in this study. These included animals that stranded alive and subsequently died or were euthanized, as well as those that washed ashore dead in good preservation status. Additionally, one animal (animal no. 11) with advanced autolysis yet compelling gross evidences of cause of death was also included. Civilian reports and regular monitoring of the coast by collaborating institutions typically initiated veterinary medical deployment followed by field autopsies. Data collected from each individual included date and location of stranding, sex, morphometrics, age class, nutritional condition and decomposition status.

Animals were divided into three age categories: calf, individuals measuring less than 7 meters of total body length and presumed to be less than 1 year of age; juvenile, 7−11.6 m in length considered to be 1 to 5 years of age; and adult, greater than 11.6 m considered to be 5 years of age or older, and sexually mature [26–30]. The nutritional status of each animal was classified as good, moderate, poor, or emaciated based on the degree of atrophy of the epaxial musculature, prominence of ribs, scapula or axial skeleton, and amount of subcutaneous, intrathoracic and abdominal fat (S1 Fig). The decomposition status was classified as fresh, moderate autolysis or advanced autolysis [31]. Descriptive epidemiologic stranding data and signalment of each individual are presented in Table 1.

**Table 1:** Individual epidemiologic stranding data and signalment of humpback whales stranded in Brazil (2004–2016).

No	Stranding date	Location (Federal Unit)	Sex	BL (m)	Age class	Stranding condition	Nutritional status	Blubber depth (cm)			Decomposition code
								D	L	V	
**1**	07-Oct-2005	Caravelas (BA)	ND	4.14	Calf	Dead	ND	NA	NA	NA	MA
**2**	17-Sep-06	Aracaju (SE)	M	4.06	Calf	Alive	Moderate	NA	NA	NA	MA
**3**	04-Aug-07	Belmonte (BA)	M	10.1	Juvenile	Alive (E)	Good	11	13.5	10.5	MA
**4**	21-Aug-07	São Mateus (ES)	M	4.37	Calf	Dead	Good	3	4	5	MA
**5**	08-Sep-07	Pirambu (SE)	F	4.87	Calf	Alive	Moderate	NA	NA	NA	MA
**6**	04-Jul-08	Alcobaça (BA)	M	3.83	Calf	Alive	Good	3.5	4.5	6	MA
**7**	14-Aug-08	Conceição da Barra (ES)	M	3.9	Calf	Alive	Moderate	3.5	3.6	3.4	MA
**8**	04-Jul-09	Guriri (ES)	F	9.18	Juvenile	Alive	Good	19	12.5	12.5	MA
**9**	21-Aug-09	Bitupita (CE)	F	10	Juvenile	Alive	Good	14.5	NA	16	Fr
**10**	08-Sep-09	São Mateus (ES)	F	4.0	Calf	Alive (E)	Moderate	3.5	6	4.5	Fr
**11**	11-Sep-09	São Mateus (ES)	F	4.4	Calf	Dead	Good	NA	NA	NA	AA
**12**	19-Aug-10	Itaporanga (SE)	F	4.63	Calf	Dead	Good	NA	NA	NA	Fr
**13**	22-Aug-10	Capão da Canoa (RS)	M	12.5	Adult	Alive	Poor	NA	NA	NA	MA
**14**	04-Sep-10	São Gonçalo do Amarante (CE)	M	3.94	Calf	Alive	Good	4.5	4	5	Fr
**15**	14-Jul-11	Balneário Pinhal (RS)	F	7.73	Juvenile	Alive (E)	Emaciated	NA	NA	NA	MA
**16**	10-Sep-11	Linhares (ES)	M	3.27	Calf	Alive	Good	3.5	3	3.5	MA
**17**	06-Sep-12	Aracaju (SE)	F	4.5	Calf	Dead	Poor	5	3	4	Fr
**18**	08-Sep-13	Barra do Riacho (ES)	M	3.5	Calf	Alive	Good	4	3	5.5	MA
**19**	30-Sep-13	Prado (BA)	M	4.0	Calf	Alive	Good	3.5	3.6	4.5	MA
**20**	10-Oct-13	Santa Cruz de Cabrália (BA)	M	4.5	Calf	Alive	Good	5	3	4	MA
**21**	09-Oct-14	Alcobaça (BA)	F	5.15	Calf	Alive	Good	6	4.5	5	MA
**22**	31-Jul-16	Porto Seguro (BA)	M	3.74	Calf	Alive	Moderate	3.8	4.3	4.5	Fr
**23**	10-Aug-16	São Mateus (ES)	M	4.27	Calf	Alive (E)	Poor	2.5	3.2	5	Fr
**24**	30-Aug-16	Linhares (ES)	M	4.54	Calf	Alive (E)	Moderate	4.5	4	3	Fr

BA—Bahia; SE—Sergipe; ES—Espírito Santo; CE—Ceará; RS—Rio Grande do Sul; M—male; F—female; ND—Not determined; BL—body length; (E)—euthanized; D–dorsal; L–lateral; V–ventral; NA–not available; Fr—fresh; MA—moderate autolysis; AA—advanced autolysis.

Autopsies were performed according to a standardized protocol [31]. Tissue collection varied depending on degree of autolysis and accessibility to the carcass so in partial autopsies tissue sampling was less extensive. Tissues were collected and fixed in 10% neutral buffered formalin, trimmed, embedded in paraffin, sectioned at 5 μm, and stained with hematoxylin and eosin (H&E) for light microscopic examination (tissues collected and evaluated histologically are recorded in S1 Table). Gram (animals no. 10, 23 and 24), and Masson’s Trichrome and Verhoeff’s (animal no. 23) stains were carried out on selected tissue sections to better characterize histopathological findings.

Immunohistochemical (IHC) labeling for *Toxoplasma gondii* and morbillivirus antigen was performed on selected tissue sections (S2 Table). Commercially available mouse monoclonal antibody solution against canine distemper virus nucleoprotein antigen (1:100; VMRD Inc, Pullman, WA) [32] and goat polyclonal anti-*T*. *gondii* antiserum solution (1:400; VMRD Inc) [33] were used as primary antibodies. Brain from a morbillivirus-positive [24] Guiana dolphin (*Sotalia guianensis*) and adrenal gland from a *T*. *gondii*-positive [34] Guiana dolphin, and tissue sections from negative Guiana dolphins were used as control tissues. Samples from blowhole and bronchial exudate were collected from animal no. 10 and from blowhole of animal no. 23 during field procedures using sterile Stuart transport medium swabs. All swabs were stored at ambient temperature and submitted to the laboratory within 24 h for routine aerobic bacterial culture techniques [35].

## Results

Twenty-four humpback whales found stranded along the Brazilian coast were subjected to a complete or partial autopsy between January 2004 to December 2016. Twenty-three whales had a sufficient quality of preservation for histopathological analysis, and in one animal, diagnosis was concluded on gross examination (animal no. 11). The 24 animals consisted of 14 (58.3%) males, 9 (37.5%) females, and one (4.2%) of unknown sex. Age distribution included 19 (79.2%) calves, four (16.7%) juveniles, and one (4.2%) adult.

### Pathologic findings and causes of stranding and/or death

**Fig 1:**
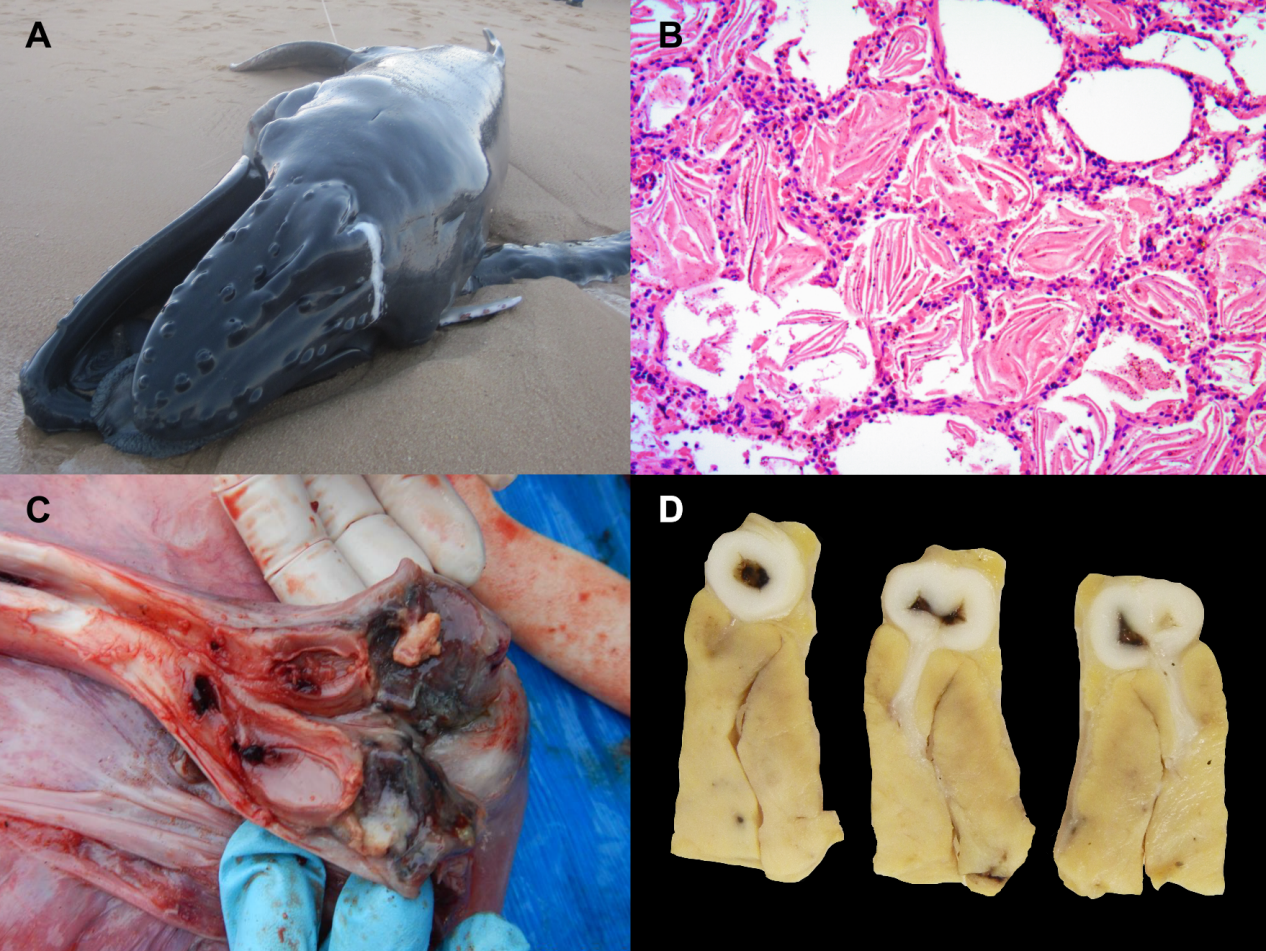
Gross and histopathologic findings in stranded humpback whales in Brazil. A) Pulmonary edema (animal no. 21). Large amounts of white foamy fluid fill in the respiratory airways and ooze trough the blowhole. B) Pulmonary parenchyma (animal no. 18). Large amounts of intra-alveolar keratin squames. C) Fibrinosuppurative umbilical arteritis (animal no. 23). The distal end of the right umbilical artery and adjacent urachus are dark red discolored and contain fibrin cast within the lumen. D) Right ventricle (animal no. 23). A major descending branch of the right coronary artery shows a markedly narrowed, 1 cm in diameter, white segment with circumferential irregularly thickened walls. Communicating with the former is a mural arterial ramification that penetrates deep into the underlying myocardium.

Main gross and histologic findings are listed following based on relevance and association with cause of stranding and/or death (CSD). Additionally, main gross and histologic findings for calves and other age categories are summarized in S3 and S4 Tables, respectively. All animals included in neonatal respiratory distress category had moderate to severe changes in the respiratory system, dominated by pulmonary edema. This was characterized by abundant white foam which in most cases filled the upper and lower airways (Fig 1A). Most significant findings in lung sections examined included: perivascular, alveolar and/or interstitial edema (19/19; 100%), and intra-alveolar or intra-bronchiolar individual squames with retained or lost nuclei, or variably-sized clusters of nucleated stratified epithelium (16/19; 84.2%) (Fig 1B). They occasionally obliterated the entire bronchiolar and alveolar lumina. Following decreasing order of occurrence in calves, we observed: atelectasis (10/19; 52.6%); hyaline membrane formation (5/19; 26.3%); and aspirated meconium (1/19; 5.2%). Findings suggestive of an inflammatory response in these animals encompassed lymphoplasmacytic interstitial pneumonia (1/19; 5.3%) and suppurative bronchopneumonia (1/19; 5.3%). Additionally, multifocal parenchymal arterial cartilaginous emboli (2/19; 10.5%) and parabronchial arterial occlusive fibrinocellular thrombosis (1/; 5.3%), were noted.

In three calves, bacterial disease was considered the CSD. All of them stranded alive and given the progressive deterioration of clinical condition and poor prognosis, humane euthanasia was elected. In animal no. 10, lesions suggestive of septicemia included suppurative bronchopneumonia with intralesional gram-negative bacilli and hemorrhage, acute hemorrhagic and neutrophilic gastroenteritis, multicentric lymphoid depletion, and multiorgan intravascular and extravascular (pulmonary alveoli) gram-negative bacillary bacterial emboli. This animal also presented pulmonary acute sand aspiration, intra-alveolar squames, edema and atelectasis; acute renal tubular necrosis; acute hepatocellular degeneration and necrosis with mild dissociation and scattered hemorrhage; and necroulcerative dermatitis. *Escherichia coli* and *A*. *hydrophila* were isolated from blowhole and bronchial exudate. In animal no. 23 and 24 (Fig 1C), omphalitis, umbilical arteritis and urachocystitis with intralesional gram-negative bacilli were accompanied by systemic congestion, hemorrhages and occasional thrombosis, along with multicentric lymphoid depletion, erythrocytosis and erythrophagocytosis. In animal no.23, two 1–2 cm in diameter, well-demarcated, pale tan nodules centered on large descending branches of the right coronary artery were noted grossly (Fig 1D). Histologically, these nodules consisted of aneurysmal coronary segments with markedly thickened walls composed of moderately disorganized tunica media and subintimal collagen and elastin bundles with proliferating myofibroblasts, along with myxedema and intimal luminal projections, as suggested by Masson’s trichrome and Verhoeff’s staining. No inflammation was observed. These histologic features were compatible with congenital coronary artery fibromuscular dysplasia [36].

Non-predatory trauma was incriminated as the CSD in two animals (animal no. 18 and 19) [37,38]. Lesions associated were: severe hemothorax and hemorrhage spanning the intercostal musculature and the parietal pleura, associated with two simple short-oblique proximal rib fractures (4^th^ and 5^th^ ribs on left side) (animal no. 18); and epidural and subarachnoid cervical hemorrhage, hemoperitoneum, and pulmonary hemorrhage and edema (animal no. 19). Animal no.11 (advanced autolysis) presented multifocal hematomas in the head, nuchal region, dorsum, right lateral flank and peduncle, and right flipper; distal amputation of the right flipper; and three deep, linear and parallel cutaneous lacerations, highly supportive of a propeller cut (vessel strike), in the left flank, caudally to the flipper and cranially to the dorsal fin. These propeller-inflicted cutaneous incisions (cranio-caudal decreasing length) involved: a 49 cm-long anterior section [regular edges, dorso-ventral orientation, slight cranio-caudal tilt, approximately 45 degrees angle with body axis] that penetrated into the abdominal cavity; an 18 cm-long, slightly more superficial middle section; and an 11 cm-long caudal incision. The last two cuts did not penetrate into the abdominal cavity. The interlaceration distance was 19 cm. Histopathologic analysis was precluded due to advanced autolysis in this individual.

Emaciation was considered the CSD in one juvenile (animal no. 15) and one adult (animal no. 13) that stranded alive. In animal no. 15, humane euthanasia was elected. Both animals presented marked atrophy of the axial skeletal musculature, most prominently in the epaxial muscles, visible and palpable bony prominences (ribs, scapula, vertebral transverse and dorsal apophyses), and marked depletion of subcutaneous, intrathoracic, intraabdominal and visceral fat depots.

**Fig 2:**
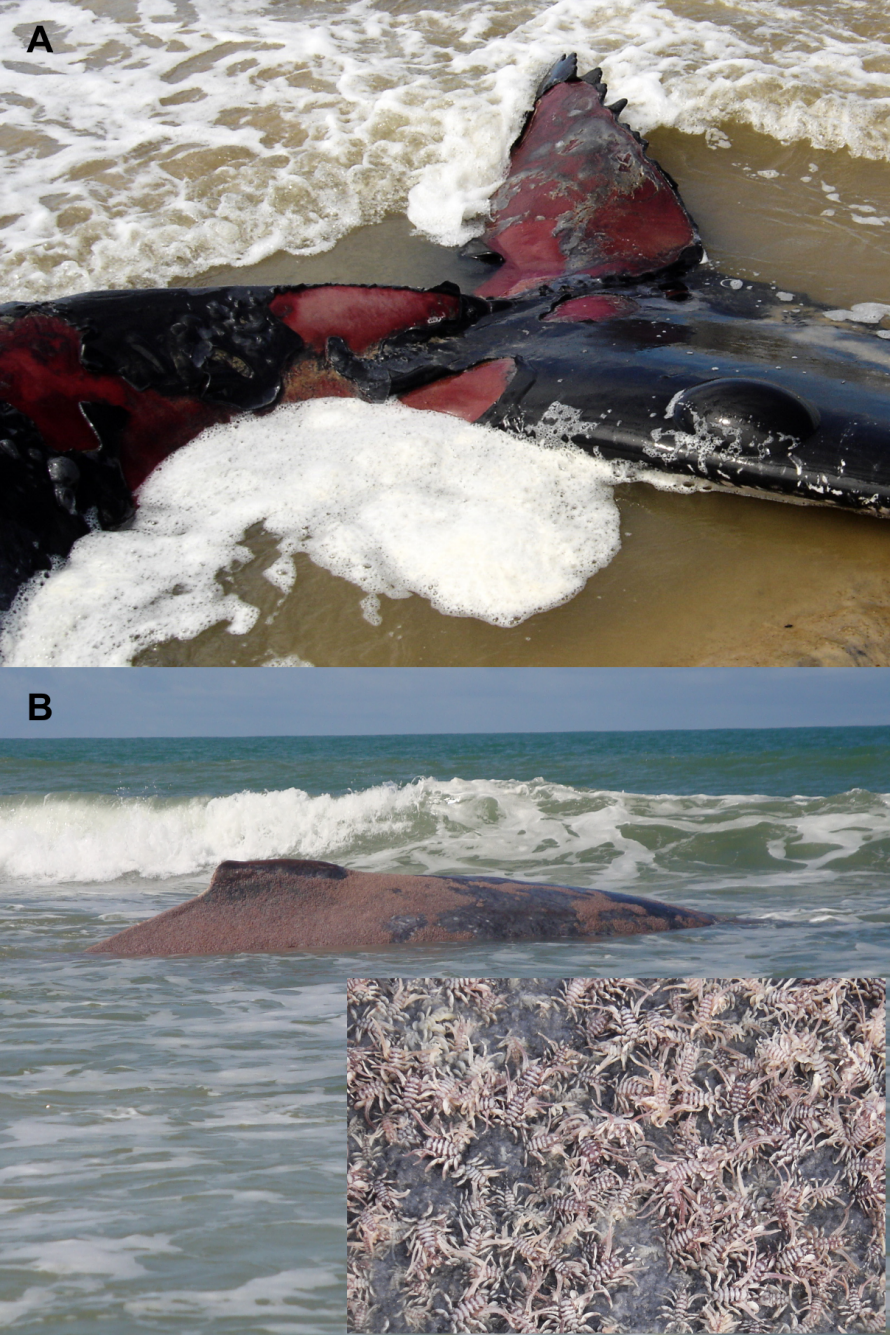
Skin lesions in stranded humpback whales in Brazil. A) Sunlight-induced thermal burn (animal no. 3). Multiple sunburn-associated bullae and blisters along with extensive areas of dermo-epidermal clefting and ulceration throughout the left lateral peduncle and dorsal aspect of the tail fluke B) Cyamid cutaneous infestation (animal no. 8). Massive cutaneous infestation by whale lice (*Cyamus* sp.) covering more than 60% of the epidermis (partially visible). Inset: Close-up view of *Cyamus boopis* cutaneous infestation (image from left flank of the animal).

In animal no. 3, severe sunlight-induced thermal burn developed (antemortem) shortly after live-stranding. Progressive solar burn manifested as extensive patchy to generalized epidermal clefting and sloughing typically associated with variably sized fluid-filled vesicles and bullae throughout the exposed dorsum and to a lesser extent along the lateral body aspects (Fig 2A and S1A Fig). Lesions progressed over a period superior to 24 hours. Given the rapidly deteriorating clinical condition, humane euthanasia was performed. Histologically, sunburn-associated lesions were characterized by severe, diffuse supra-basilar to dermo-epidermal clefting and marked superficial dermal necrosis. Additional relevant findings included pulmonary edema and hemorrhage and systemic congestion with scattered hemorrhage.

Massive cutaneous infestation by whale lice (*Cyamus boopis*) was observed in animal no. 8, covering more than 60% of the body (Fig 2B). On autopsy, this animal had severe caudal discospondylitis characterized by erosions and cavities on the articulating epiphyseal surfaces of two caudal vertebrae with a thick layer of exuberant exostosis along the entire circumference of the vertebral bodies [specimen #333, published in [23]].Histologic analysis from this tissue was not available. Discospondylitis was considered responsible for reduced locomotion and eventual CSD.

Interspecific interaction, i.e., shark attack, was the most frequent gross finding observed in 10/24 (41.7%) cases, which included multifocal, round to oval (3 to 7 cm in a major axis)[39,40], deep cutaneous wounds highly compatible with cookiecutter shark bites (*Isistius* spp.), in most cases. The animal in animal no. 5 had a large deep semi-lunar shaped wound, approximately 23 cm of circumference [41,42], compatible with a tiger shark (*Galeocerdo cuvier*) bite [S2 Fig]. Shark bites were considered incidental due to minimal severity and extent except in animal no.5.

Other gross findings worth noting in calves/neonates are summarized as follows. Four animals (animals no.12, 17, 21 and 23) had single or scattered superficial linear skin abrasions and lacerations, suggestive of net markings. From these, two animals stranded alive and two stranded dead. Three animals (animals no. 6, 14 and 24) presented small to moderate amounts of milk in the stomach. Twelve of 19 (63.1%) calves presented incomplete umbilical healing (including three animals with infectious disease as CSD), while 2/19 (10.5%) presented incomplete healing, as suggested by presence of irregular, pedunculated and reddened remnants of the umbilical cord or opened umbilical scar (S3 Fig). In five (26.3%) calves, the umbilicus was not evaluated due to logistic limitations. Fetal folds, noted mostly in the ventral and lateral aspect of the peduncle were seen in 13/15 (86.6%) and furled dorsal fin was observed in 6/14 (42.8%).

Similarly, worth noting histologic findings are described as follows (Table 2). Acute skeletal and cardiac myocyte degeneration (as suggested by cytoplasmic hypereosinophilia, angulosity and hyperchromatic nuclei) with or without contraction band necrosis (9/21; 42.8%), acute renal tubular necrosis (7/21; 33.3%) and intracytoplasmic hepatocellular hyaline globules with ‘pink points’ and ‘eosinophilic fibrils’ (12/21; 57.1%), were observed in our study, predominately in animals that stranded alive. All calves presented varying degrees of hepatocellular vacuolar change (lipid type). In the hematopoietic system, morphologic evidences of immunosuppression, as suggested by splenic lymphoid depletion (7/15; 46.7%) and multicentric nodal lymphoid depletion (7/11; 63.6%), were observed. Furthermore, extramedullary hematopoiesis characterized by immature (2–4 fused nuclei) megakaryocytes with variable numbers of myeloblastic and lymphoblastic precursors was observed in all spleens evaluated (n = 15), and occasionally in lymph nodes (6/11; 54.5%), liver (6/21; 28.6%), thymus (2/8; 25%) and lung (5/21; 23.8%). Morbilliviral and *Toxoplasma gondii* antigen testing by IHC analysis was negative in all the animals assessed (n = 19).

**Table 2:** Summary of the main histopathologic findings in 23 humpback whales* stranded in Brazil (2004–2016).

Organ or System	Main histopathological findings	Total of affected animals	Total examined	% of total
Lung	Pulmonary edema (perivascular, alveolar and/or interstitial) with/without alveolar histiocytosis	19	21	90.5
	Intra-alveolar or intra-bronchiolar epithelial squames	16	21	76.2
	Atelectasis	10	21	47.6
	Hyaline membrane formation	5	21	23.8
	Extramedullary hematopoiesis	5	21	23.8
	Lymphoplasmacytic interstitial pneumonia	3	21	14.3
	Lymphocytic bronchitis	3	21	14.3
	Arterial cartilaginous emboli	2	21	9.5
	Suppurative bronchopneumonia with type II pneumocyte hyperplasia	1	21	4.8
	Arterial occlusive fibrinocellular thrombosis	1	21	4.8
	Aspirated meconium	1	21	4.8
Heart	Acute myodegeneration with/without contraction band necrosis	9	21	42.8
	Hemorrhage	7	21	33.3
	Lymphoplasmacytic and neutrophilic myocarditis	1	21	4.8
	Congenital coronary artery fibromuscular dysplasia	1	21	4.8
Rete mirabile	Multifocal thrombosis and interstitial hemorrhage	1	4	25
Brain and/or spinal cord	Leptomeningeal and parenchymal congestion	5	5	100
	Leptomeningeal and/or neuroparenchymal hemorrhage	4	5	80
	Perivascular, myelinic and cytotoxic edema	1	5	20
Liver	Hepatocellular vacuolar change (lipid type)	20	21	95.2
	Intracytoplasmic hepatocellular hyaline globules with ‘pink points’ and ‘eosinophilic fibrils’	12	21	57.1
	Extramedullary hematopoiesis	6	21	28.6
	Hepatocellular degeneration and necrosis	5	21	23.8
	Hemorrhage	3	21	14.3
	Periportal (centroacinar) lymphoplasmacytic infiltrates	3	21	14.3
	Bile duct hyperplasia	2	21	9.5
	Cholestasis	1	21	4.8
Stomach	Lymphoplasmacytic and neutrophilic gastritis	4	18	22.2
	Acute apical hemorrhage with erosion and ulceration	2	18	11.1
Intestine	Superficial hemorrhagic enteritis	1	18	5.5
	Lymphoplasmacytic and eosinophilic enteritis	1	18	5.5
Tongue	Ulcerative and neutrophilic glossitis with intralesional bacteria and thrombosis	1	14	7.14
Kidney	Acute tubular necrosis	7	21	33.3
	Acute capsular hemorrhages	7	21	33.3
	Interstitial lymphoplasmacytic infiltrates	2	21	9.5
	Mesangiocapillary glomerulopathy	2	21	9.5
	Tubular ectasia with proteinosis and epithelial attenuation	2	21	9.5
Urinary bladder with/without urachus	Urachocystitis	2	14	14.3
Umbilical cord	Subacute omphalitis/umbilical arteritis with/without intralesional bacteria	2	3	66.6
Genital	Perimetritis with multifocal hemorrhage	1	7	14.3
	Acute testicular hemorrhage	1	7	14.3
Spleen	Extramedullary hematopoiesis	15	15	100
	Lymphoid depletion	7	15	46.7
	Acute subcapsular hemorrhages	2	15	13.3
	Lymphocytolysis	1	15	6.7
	Thrombosis	1	15	6.7
Lymph nodes	Multicentric lymphoid depletion	7	11	63.6
	Extramedullary hematopoiesis	6	11	54.5
	Sinus histiocytosis with/without erythrocytosis	5	11	45.4
	Follicular hyalinosis	1	11	9.1
	Lymphoid reactive hyperplasia	1	11	9.1
Adrenal gland	Focal adrenal arterial cartilage embolus	1	7	14.3
Thymus	Extramedullary hematopoiesis	2	8	25
Integument	Intercellular edema	5	17	29.4
	Dermal hemorrhage	4	17	23.5
	Dermo-epidermal separation	4	17	23.5
	Focally extensive necroulcerative dermatitis and bacteria with/without thrombosis	3	17	17.6
	Generalized neutrophilic dermatitis with necrosis, bacteria and cyamids	1	17	5.9
	Suppurative epidermitis	1	17	5.9
Skeletal muscle	Lymphoplasmacytic and neutrophilic myositis	1	13	7.7
	Acute segmental myocyte degeneration	1	13	7.7

*Animal no.11 (vessel strike) is not included.

The most probable cause of stranding and/or death (CSD) was determined in 23/24 individuals (95.8%; Table 3). In calves, these included neonatal respiratory distress (along with maternal separation) in 13 of 19 calves (68.4%), infectious disease (septicemia, omphaloarteritis and urachocystitis; 3/19; 15.8%), trauma of unknown origin (2/19; 10.5%), and vehicular trauma (vessel strike; 1/19; 5.3%). Neonatal respiratory distress occurred concomitantly with trauma of unknown origin in two cases (animals no.18 and 21) and antemortem predation by sharks in one case (animal no. 5). In animals no. 23 and 24, infectious omphalitis, umbilical arteritis and urachocystitis coexisted with neonatal respiratory distress. In juveniles and adult individuals, CSD were: emaciation (2/5; 40%), sunlight-induced thermal burn injury (1/5; 20%); and discospondylitis (1/5; 20%). In one juvenile, the CSD was not determined (1/5; 20%).

**Table 3:** Summary of most probable causes of stranding and/or death of 24 humpback whales stranded in Brazil (2004–2016).

Cause of stranding and/or death	Age class			Partial total	% of total
	Calf	Juvenile	Adult		
Neonatal respiratory distress	13	0	0	13	54.1
Infectious disease	3	1	0	4	16.7
Trauma (unknown origin)	2	0	0	2	8.3
Emaciation	0	1	1	2	8.3
Sunlight-induced thermal burn	0	1	0	1	4.2
Trauma (vessel strike)	1	0	0	1	4.2
Not determined	0	1	0	1	4.2

Bacteriology on the blowhole swab and bronchial exudate yielded *Escherichia coli* in pure culture and *E*. *coli* and *Aeromonas hydrophila*, respectively, in animal no. 10. *Pseudomonas aeruginosa* was isolated from blowhole swab of animal no. 23.

## Discussion

In this study we examined 24 dead humpback whales found stranded in the coastline of Brazil. All regions (northeast, southeast and south) where this species is known to inhabit were represented; a total of 608 animals were recorded to strand between 2004 and 2016 (Humpback Whale Institute-Brazil, personal communication). Most animals were found in the Abrolhos bank, the main breeding area for humpback whales in the South-western Atlantic Ocean. As expected, the pathologic condition most frequently encountered involved neonatal or perinatal disease in which morphologic and etiologic characteristics are typically related to possible alteration in birth, nursing or behaviour [43]. Neonatal or perinatal pathology in cetaceans encompass a constellation of etiologic diagnoses including: abortion, dystocia, early maternal-filial separation or maternal neglect, early fatal intra- and interspecific interactions, infections, loss of passive transfer immunity, prematurity, and congenital malformations, among others [43–45]. Disturbances during gestation, delivery, lactation and early behavioral abnormalities may be also included [43].

In this study, consistent intrabronchiolar and intra-alveolar squamous epithelial cells (keratin squames) and moderate to severe edema along with variable formation of hyaline membranes was suggestive of acute respiratory distress. Additionally, one animal also presented meconium aspiration. While some studies in human and animals have shown that meconium aspiration and keratin squames are not a normal consequence of birth [46], others refute pathologic significance of keratin squames [47]. In the present study, moderate to severe edema was considered the feature with major pathological significance, in agreement with previous studies focused on acute lung injury (ALI) [46,48,49]. The pathogenic mechanisms involved in these cases are obscure; however, major consideration is given to increased vascular permeability and increased hydrostatic pressures, as previously suggested [48,49]. The possibility that pulmonary edema ensues partially and aggravates the pathological picture as an “agonal” or “terminal” event cannot be completely ruled out in these cases. Hyaline membrane formation is a consistent yet non-specific feature of ALI and a major component of neonatal respiratory distress syndrome (NRDS), so called ‘hyaline membrane disease’ in humans and its recognized animal counterpart [47]. Deficiency of pulmonary surfactant, the fundamental defect in NRDS (typically ascribed to SFTPB and SFTBC genes defect), remains to be demonstrated in cetaceans. Concentric hyaline membranes have been seen occluding the bronchioles of newborn harbor porpoises[50] and a striped dolphin (*Stenella coeruleoalba*) [45]. Recently, a case compatible with meconium aspiration syndrome was described in an Atlantic bottlenose dolphin [51] that died immediately after birth and a newborn harbour porpoise (*Phocoena phocoena*) [50]. In the former, a knot in the umbilical cord might had led to hypoxia [51]. Our results suggest that perinatal asphyxia or respiratory distress appears to occur quite frequently and should be taken into account as a possible cause of neonatal mortality in this humpback whale population.

Two other important conditions listed under neonatal/perinatal pathology in cetaceans that could not be determined in this study include dystocia and prematurity. A diagnosis of dystocia is facilitated by visual recognition (e.g., in captive animals or in free-ranging dams developing labor in the coast). If visual contact is not attained (vast majority of field situations), a diagnosis of dystocia may hinge on identification of areas of circumferential to linear bilateral congestion and hemorrhage in mandibular, cranial, thoracolumbar, pectoral flippers, and abdominal wall, potentially related to uterine contraction or birth canal pressure [45]. Autopsy examination should confirm these gross findings, and histologic analysis may reveal similar respiratory distress-associated changes above described. Nonetheless, distinguishing fatal dystocia from a potential non-lethal dystocic or non-dystocic birth followed by neonatal weakness, traumatic interaction and subsequent mother-calf separation or calf-neglect may prove troublesome.

Prematurity was considered as a major pathologic condition in common bottlenose dolphins [52]. Prematurity is the second most common cause of neonatal mortality after congenital abnormalities in humans [53]. Main risk factors for prematurity include: premature rupture of placental membranes; intrauterine infection; uterine, cervical and placental structural abnormalities; and multiple gestation [53]. A diagnosis of prematurity in humpback whales or any other cetacean species for which there is insufficient life history (e.g., period of gestation of the damn) and morphologic (e.g., total body length at birth [BLB]) data to stablish prematurity, should be one of exclusion. In these cases, gross and histological analyses should rule in hypoplasia or incomplete patterns of maturation of single or multiple organs. Currently, there is no consensus on BLB for this species, and scientific literature from whaling operations or stranded carcasses from different geographical locations, reveal heterogeneity (4.5–5 m[54]; 3.9–4.3 m[26]). In the present study, we observed completely healed umbilici in animals measuring down to 3.9 m-long (animal no.7), in contrast to animals with incomplete umbilical healing that measured up to 5.15 m-long (animal no. 21). Irrespective of intervening biological variables, our results suggest individual intraspecific variations for BLB in neonates of the same population. Further studies with greater sample size are required for better evaluation of potential of BLB as an indicator of prematurity. In this regard, the authors have observed that large distal segments of umbilical arteries, running parallel to the urinary bladder towards the umbilical ring, were not occluded by fibrin clots, as normally observed in other cetacean species shortly after birth. We believe the degree of physiological umbilical arterial fibrin occlusion and regression in cetaceans merit further investigation before any conclusion is drawn. Furthermore, we did not observe any gross or histological evidence of dysmaturity or immaturity that would support a diagnosis of prematurity in any of these animals.

Newborns are susceptible to infections, especially premature animals or individuals with delayed development [53]. Infections may be acquired in utero, during birth or perinatally. Lung involvement is common [53]. In utero, fetal transmission may occur via ascending mechanisms or transplacentally, through the maternal bloodstream [53]. Several animals in this study presented evidence of neonatal/perinatal infection. In animal no. 10, pathologic findings coupled with bacteriology results were suggestive of a systemic infectious process where *E*. *coli* and *A*. *hydrophila* might have played a major role leading to septicemia. Reduced transfer or absorption of maternal colostral immunoglobulins might have been a predisposing factor; however, no determination of IgG was performed in this animal. Since *E*. *coli* was cultured from the blowhole and bronchial exudate, it is possible that the pathologic agent entered via the respiratory tract. No other organs were cultured. In animals no. 23 and 24, omphalitis, umbilical arteritis and urachocystitis with intralesional bacteria were accompanied by systemic congestion, hemorrhages and occasional thrombosis, along with multicentric nodal and splenic lymphoid depletion, erythrocytosis and erythrophagocytosis. In both animals, the etiologic agent remains unknown. The pathologic significance of *Pseudomonas aeruginosa* isolated from blowhole of animal no. 23 is uncertain. Further research is needed to investigate infectious etiologies in newborns in this population and in those were higher mortality rates of young individuals are reported.

Mating and courtship activity involving numerous males and a female, often accompanied by a newborn, have been observed in the Abrolhos bank region [55] and is a potential source of trauma in humpback whales. Animals no.18 and 19 (calves) had evidences of multiple trauma possibly due to intense intra-specific interaction, as suggested by recent superficial skin lacerations running in multiple directions and associated subcutaneous edema and hemorrhage. In animal no. 18, multiple fractured ribs and systemic hemorrhage were noted. In animal no. 21 (calf), although the cause of death was largely attributed to respiratory distress, the animal also presented focal hemorrhage in the dorso-cervical region and multifocal parenchymal arterial cartilaginous emboli, suggesting antemortem trauma. No bone fractures were observed. Presumptive trauma in this case was considered a precipitating event for stranding.

Anthropogenic interactions such as vessel strikes and entanglement in fishing gear have been reported sporadically in some humpback whale populations [8,9,11,12,14–16]. In our study, vessel strike was the cause of death of 1/24 animals (animal no.11) while evidences of net markings, suggestive of entanglement, were observed in 4/24 individuals. Two of the animals (animal no. 21 and 23) stranded alive, lending support to sublethal entanglement. Our interpretation suggests entanglement in these cases could have precipitated stranding. Death was attributed to neonatal respiratory distress (animals no.12 and 17), trauma (animal no. 21) and septicemia (animal no.23), and entanglement could not be ruled out as a concomitant condition. The presence of equivocal (often mimicked by intra- and interspecific markings or excoriations associated with live-stranding events) or unequivocal net markings should prompt consideration for entanglement. Diagnosticians may rule out other CSD, yet net entanglement should be included in the list of differential etiologic diagnoses for stranding and death in this species worldwide.

In a study of 38 humpback whales stranded in the eastern cost of US between 1985 and 1992, the death of six animals (30%) was attributed to ship strike, while five (25%) were related to entanglement in fishing gear. One animal presented wounds and marks compatible with both conditions [16]. Vessel strike was the cause of death in 1/19 humpback whales recorded between 1972 and October 1996 in the main Hawaiian Islands [15]. In Colombia, from 24 strandings registered between 1986 and 2000, ten whales were entangled in fishing gears and three were killed by vessel strikes [12].

Emaciation has been occasionally reported in whales. Starvation was determined to be the cause of death of two humpback whales in the United Kingdom between 2005 and 2010 [56]. An extremely malnourished state as evidenced by severe generalized adipose hypoplasia was observed in 2/3 neonates autopsied in Western Australia in 2011 [57]. In our study, a severely emaciated adult (animal no.13), had lymphoplasmacytic bronchitis and *Vibrio mediterranei* was isolated from the right lung (specimen #1409, published in Moura et al.) [22]. Additionally, a juvenile whale (animal no.15) was found severely emaciated without manifest evidence of the underlying cause. Causes of emaciation in migratory mysticete species are sometimes not readily evident at autopsy or histopathological examination. Consideration should be given to issues concerning health, behavior or food availability in feeding grounds before migration is initiated [58]. Blubber measurements could serve as an objective indicator for determination of the nutritional status in this species, however the intra-observer variations and measuring at slightly divergent anatomical locations (due to restricted carcass handling) precluded the delineation of blubber thickness’ ranges by age class.

In this study, animal no. 3 represented an extreme example of sunlight-induced thermal burn. The animal, which stranded alive with intact epidermis, developed a progressive locally extensive, patchy to generalized epidermal clefting and sloughing associated with variably sized, fluid-filled vesicles and bullae, recapitulating findings observed in severe sunlight and photochemical skin exposure cases [59–62]. We believe the severity and extent of the lesions in this case likely resulted in severe homeostatic disturbances similar to those identified in thermal burn shock along with hyperthermia and alarm response [59,63,64], leading to medical decision of euthanasia in this animal. The clinicopathological features of this pathologic process in cetaceans merit further investigation. Thermal injury is known to induce severe acute changes in living tissues yet the associated pathogenesis of the fluid shifts with subsequent edema formation and fluid losses is not fully understood [59]. Visible swelling of the skin, blister formation, and loss of surface-protecting epithelium are among the typical clinical features [59,62,63]. With these shifts and losses of fluid from the circulation, hypovolemia develops and if appropriate fluid therapy is not instituted, hypovolemic shock ensues [59,63]. Additional clinical complications may include obstruction of the venous and lymphatic return, severely reduced arterial blood supply and restriction of respiratory movements (by thoracic burns) [63]. In human medicine, surface area injured is more important for outcome than is depth [61,65]. Human patients often describe local heat, tingling sensation, and sometimes excruciating pain of the bullous lesions [65].

In animal no. 8, massive cutaneous infestation by whale lice (*Cyamus boopis*) was observed covering more than 60% of the body. On autopsy, this animal presented severe caudal discospondylitis. Despite cyamids feed on host’s skin (typically in areas with lowest turbulence of water flow, e.g., around barnacles, skin folds or ventral grooves of the head, protected zones around the blowholes, eyes, and flippers, margins of the lips, on callosities, wounds, and genital slit), they appear to bear no pathological relevance [66]. It has been hypothesized, higher burdens of cyamids may be a consequence of reduced host locomotion [66]. In the present case, discospondylitis was conceivably responsible for reduced locomotion, what might have favoured intense cyamid colonization, and eventual stranding.

Shark bites were the most frequent gross finding observed in 11/24 (45.8%) animals. Shark bites were considered incidental because of limited severity and extent in most of the cases. However, multiple shark attacks likely propitiated live-stranding event in animal no. 5, and might aggravated or played a major role in development of respiratory distress. The basis for shark bite diagnosis hinges on the curvature of the wounds and distance/shape of incisive marks. Predation and aggression by sharks or other cetacean species have been observed in humpback whales in Hawaiian waters and in the region of Queensland, Australia [15,67,68], and should be considered as a cause of stranding and/or death in cetacean species worldwide.

Death of live-stranded cetaceans occurring in the absence of grossly evident pathologic conditions has been often attributed to a catecholaminergic shock or ‘alarm reaction’ coupled with musculoskeletal and variable visceral damage resembling capture myopathy [64,69]. Acute skeletal and cardiac myocyte degeneration with/without contraction band necrosis, acute renal tubular necrosis, intracytoplasmic hepatocellular hyaline globules with ‘pink points’ and ‘eosinophilic fibrils’, and acute centrilobular hepatocellular degeneration, dissociation and sinusoid congestion were commonly observed in live-stranding animals. These findings are in agreement with previous observations [64,69,70]. Despite their non-specific nature, they prompt consideration of a live-stranding event. Hepatocellular hyaline globules were also a common finding (12/16), variably accompanied by acute centrilobular hepatocellular degeneration, hepatocellular dissociation and sinusoid congestion. Most animals of the present study (18/24; 75%) stranded alive and likely underwent hemodynamic disturbances compatible with a stress response, as suggested by gross and histologic findings. Again, this change is commonly seen in live-stranded cetaceans.

All calves presented variable degrees of hepatocellular vacuolar (lipid type) change. This is a common feature in the liver of neonates and young calves of many other mammal species. In absence of any association with further liver pathologic findings, it was interpreted as a physiologic finding, and likely to be associated with fasting.

Cowan and Smith [71] found megakaryocytes in the spleens of all 50 examined bottlenose dolphins, suggesting that hematopoiesis takes place in the spleen in that species. In our study, all examined spleens presented megakaryocytes and blast cells of the myeloid, erythroid and lymphoid lineage. They were also occasionally observed in the liver, thymus, lung and lymph nodes. This finding supports the hypothesis that extramedullary hematopoiesis is a physiologic finding due to limited bone marrow reserves in this species.

All animals that stranded alive were repeatedly hit by waves at tidal line for varying periods of time during medical assistance. Only few of them presented compelling gross or histologic evidences of water inhalation, as suggested by presence of sand grains, algae and other eukaryotic organisms of the water column. In all these cases, the participation of water aspiration in mortality was deemed neglectable and a direct consequence of live-stranding. We believe water aspiration might have further aggravated preexisting clinicopathological conditions in some of the animals. A diagnosis of death by water aspiration or ‘wet drowning’ in live-stranding events requires continuous monitoring of the animals and discarding other causes of death through pathologic examination.

The susceptibility of mysticetes to morbillivirus infection has been confirmed in two fin whale (*Balaenoptera physalus*) calves [72] and one adult. The latter had concomitant *T*. *gondii* and dolphin morbillivirus infection [73]. In humpback whales, serological evidence of exposure and RT-PCR CeMV genetic material have been detected in one animal from the Gulf of Maine, US [74] and one animal from Hawaii [75], respectively. The recent report of CeMV infection in a Guiana dolphin from the coastal waters of Abrolhos Bank [24] and recent confirmed cases of toxoplasmosis in Brazil [34], prompted IHC investigation for these agents. Testing for both etiologies was negative.

In 2012, we reported skeletal pathology in stranded humpback whales where the specimens generally exhibited advanced decomposition. This study constitutes the first health survey of humpback whales along the Brazilian coast. The findings are valuable to the understanding of baseline disease processes in humpback whales. It is expected that these results will help to guide stranded animal autopsy examination and population management decisions in the future.

## Supporting information

S1 Fig Panel of nutritional status (NS) categories in humpback whales stranded in Brazil. A) Good NS, juvenile (animal no. 3). Epaxial musculature development is appropriate for the age class and fat depots are abundant (not visible), giving a convex dorsal profile. Additionally, this animal has disseminated cutaneous blisters and severe extensive ulcerated areas (sunlight-induced thermal burn; picture taken immediately after euthanasia). B) Moderate NS, calf (animal no. 7). Epaxial muscle development and fat deposits are regarded as normal for the age class, giving a slightly convex or rather straight dorsal profile. C) Poor NS, calf (animal no. 17). Epaxial muscle is mildly decreased and fat depots are reduced (not visible), giving a mild concave dorsal profile. Furthermore, costal and nuchal bony protuberances are slightly prominent. D) Emaciated NS, juvenile (animal no. 15). Epaxial muscle is markedly reduced giving a profound concave dorsal profile. Additionally, costal, nucal and transverse apophyses are prominent and fat depots are markedly depleted or unapparent (not visible).
(TIF)

S2 Fig Panel of antemortem shark bites in a humpback whale (animal no. 5) stranded in Brazil. All pictures were taken while the animal was still alive. A) and B) Cutaneous coockiecutter shark (*Isistius* spp.) with exposure of hyperemic and inflamed dermis and blubber. Bar: 1 cm. C) Cutaneous coockiecutter shark bite with exposed dermis and blubber. This picture is of particular interest because had not this animal being alive, this bite would likely be conservatively considered inflicted post mortem due to insufficient evidences (i.e., hyperemia, hemorrhage, edema, inflammatory exudate). Hence, antemortem bites are likely to be underdiagnosed in regular cetacean autopsies. Bar: 1 cm. D) Cutaneous tiger shark (*Galeocerdo cuvier*) bite with exposed deep dermis. Similarly to S2C Fig, unequivocal evidences of antemortem wounding are lacking in this bite. Bar: 5 cm.
(TIF)

S3 Fig Different degrees of umbilical cord regression in humpback whales. For both images, cranial direction is to the right. A) Umbilical cord (animal no. 12). Presence of irregular, pedunculated and reddened remnants of the umbilical cord. B) Umbilical cord (animal no. 7). The umbilical cord is completely and homogeneously healed.
(TIF)

S1 Table Tissues evaluated histologically in each case. (DOCX)

S2 Table Organs tested via immunohistochemistry for morbillivirus and *Toxoplasma gondii* antigens. (DOCX)

S3 Table Main gross and histologic findings in calf humpback whales stranded in Brazil (2004–2016). (DOCX)

S4 Table Main gross and histologic findings in juvenile and adult humpback whales stranded in Brazil (2004–2016). (DOCX)

## References

[1] Clapham PJ. Humpback whale, *Megaptera novaeangliae* In: Perrin WF, Wursig B, Thewissen JGM, editors. Encyclopedia of marine mammals. 2nd ed San Diego, CA: Academic Press; 2009 pp. 582–5.

[2] Zerbini AN, Andriolo A, Heide-Jorgensen MP, Pizzorno JL, Maia YG, Vanblaricom GR, et al Satellite-monitored movements of humpback whales *Megaptera novaeangliae* in the Southwest Atlantic Ocean. Mar Ecol Prog Ser. 2006; 313: 295–304.

[3] Engel MH, Martin AR. Feeding grounds of the western South Atlantic humpback whale population. Mar Mammal Sci. 2009; 25 (4): 964–9.

[4] Andriolo A, Martins CCA, Engel MH, Pizzorno JL, Más-Rosa S, Freitas A, et al The first aerial survey to estimate abundance of humpback whales (*Megaptera novaeangliae*) in the breeding ground off Brazil (Breeding Stock A). J Cetacean Res Manag. 2006; 8 (3): 307–11.

[5] Martins CCA, Morete ME, Engel MH, Freitas AC, Secchi ER, Kinas PG. Aspects of habitat use patterns of humpback whales in the Abrolhos Bank, Brazil, breeding ground. Mem Queensl Mus. 2001; 47 (2): 563–70.

[6] Andriolo A, Kinas PG, Engel MH, Martins CCA, Rufino AM. Humpback whales within the Brazilian breeding ground: distribution and population size estimate. Endanger Species Res. 2010; 11 (3): 233–43.

[7] Bortolotto GA, Danilewicz D, Andriolo A, Secchi ER, Zerbini AN. Whale, whale, everywhere: increasing abundance of western South Atlantic humpback whales (*Megaptera novaeangliae*) in their wintering grounds. PLoS One. 2016; 11 (10): e0164596 doi: 10.1371/journal.pone.0164596 27736958 10.1371/journal.pone.0164596 PMC5063365

[8] Hill AN, Karniski C, Robbins J, Pitchford T, Todd S, Asmutis-Silvia R. Vessel collision injuries on live humpback whales, Megaptera novaeangliae, in the southern Gulf of Maine. Mar Mammal Sci. 2017; 33 (2): 558–73.

[9] Alava JJ, Barragan MJ, Castro C, Carvajal R. A note on strandings and entanglements of humpback whales *(Megaptera novaeangliae)* in Ecuador. J Cetacean Res Manag. 2005; 7 (2): 163–8.

[10] Bogomolni AL, Pugliares KR, Sharp SM, Patchett K, Harry CT, Larocque JM, et al Mortality trends of stranded marine mammals on Cape Cod and southeastern Massachusetts, USA, 2000 to 2006. Dis Aquat Organ. 2010; 88 (2): 143–55. doi: 10.3354/dao02146 20225675 10.3354/dao02146

[11] Felix F, Haase B, Davis JW, Chiluiza D, Amador P. A note on recent strandings and bycatches of sperm whales (*Physeter macrocephalus*) and humpback whales (*Megaptera novaeangliae*) in Ecuador. Report of the International Whaling Commission 1997. 917–9 p.

[12] Capella Alzueta J, Florez-Gonzalez L, Fernandez PF. Mortality and anthropogenic harassment of humpback whales along the Pacific coast of Colombia. Mem Queensl Mus. 2001; 47 (2): 547–53.

[13] Camphuysen KCJ. Foraging humpback whale (*Megaptera novaeangliae*) in the Marsdiep area (Wadden Sea), May 2007 and a review of sightings and strandings in the southern North Sea, 2003–2007. Lutra. 2007; 50 (1): 31–42.

[14] Scheidat M, Castro C, Denkinger J, Gonzalez J, Adelung D. A breeding area for humpback whales (*Megaptera novaeangliae*) off Ecuador. J Cetacean Res Manag. 2000; 2 (3): 165–71.

[15] Mazzuca L, Atkinson S, Nitta E. Deaths and entanglements of humpback whales, *Megaptera novaeangliae*, in the main Hawaiian Island, 1972–1996. Pac Sci. 1998; 52 (1): 1–13.

[16] Wiley DN, Asmutis RA, Pitchford TD, Gannon DP. Stranding and mortality of humpback whales, *Megaptera novaeangliae*, in the mid-Atlantic and southeast United States, 1985–1992. Fish Bull. 1995; 93 (1): 196–205.

[17] Geraci JR, Anderson DM, Timeri RJ, Aubin DJS, Early GA, Prescott JH, et al Humpback whales (*Megaptera novaeangliae*) fatally poisoned by dinoflagellate toxin. Can J Fish Aquat Sci. 1989; 46 (11): 1895–8.

[18] Kogure K, Tamplin ML, Simidu U, Colwell RR. A tissue culture assay for tetrodotoxin, saxitoxin, and related toxins. Toxicon. 1988; 26: 191–7. 3363566 10.1016/0041-0101(88)90171-7

[19] Barros NB. Recent cetacean records for southeastern Brazil. Mar Mammal Sci. 1991; 7 (3): 296–306.

[20] Santos-Neto E, Rossi-Santos MR, Baracho CG, Cipolotti SR, Sampaio CLS, Velozo RS, et al A case study of a lone humpback whale calf (*Megaptera novaeangliae*) inside Baía de Todos os Santos, Bahia State, north-eastern Brazil, with implications for rescue procedures. J Mar Biol Assoc U K 2: Biodiversity Records. 2008; 1: e97.

[21] Bortolotto GA, Kolesnikovas CKM, Freire AS, Simões-Lopes PC. Young humpback whale *Megaptera novaeangliae* feeding in Santa Catarina coastal waters, Southern Brazil, and a ship strike report. Marine Biodiversity Records. 2016; 9: 29.

[22] Moura JFD, Rodrigues DDP, Roges EM, Souza RLD, Ott PH, Tavares M, et al Humpback whales washed ashore in southeastern Brazil from 1981 to 2011: stranding patterns and microbial pathogens survey. Biologia. 2013; 68 (5): 992–9.

[23] Groch KR, Marcondes MC, Colosio AC, Catao-Dias JL. Skeletal abnormalities in humpback whales Megaptera novaeangliae stranded in the Brazilian breeding ground. Dis Aquat Organ. 2012; 101 (2): 145–58. doi: 10.3354/dao02518 23135142 10.3354/dao02518

[24] Groch KR, Colosio AC, Marcondes MC, Zucca D, Diaz-Delgado J, Niemeyer C, et al Novel cetacean morbillivirus in Guiana dolphin, Brazil. Emerg Infect Dis. 2014; 20 (3): 511–3. doi: 10.3201/eid2003.131557 24565559 10.3201/eid2003.131557 PMC3944878

[25] Meirelles ACO, Campos TM, Marcondes MCC, Groch KR, Souto LRA, Reis MSS, et al Reports of strandings and sightings of bottlenose dolphins (Tursiops truncatus) in northeastern Brazil and Brazilian oceanic islands. Latin American Journal of Aquatic Mammals. 2016; 11 (1–2): 178–90.

[26] Nishiwaki M. Humpback whales in Ryukyuan waters. Sci Rep Whales Res Inst. 1959; 14: 49–87.

[27] Rice DW. Progress report on biological studies of the larger cetaceans in the waters off California. Norsk Hvalfangst Tidende. 1963; 52 (7): 181–7.

[28] Clapham PJ. Maturational changes in patterns of association in male and female humpback whales, *Megaptera novaeangliae*. J Zool. 1994; 234 (2): 265–74.

[29] Chittleborough RG. Determination of age in the humpback whale *Megaptera nodosa* (Bonaterre). Aust J Mar Freshw Res. 1959; 10 (2): 125–43.

[30] Clapham PJ. Age at attainment of sexual maturity in humpback whales, *Megaptera novaeangliae*. Can J Zool. 1992; 70 (7): 1470–2.

[31] Geraci JR, Lounsbury VJ. Marine Mammals Ashore: a field guide for strandings. Baltimore, MD: National Aquarium in Baltimore; 2005.

[32] Saliki JT, Cooper EJ, Gustavson JP. Emerging morbillivirus infections of marine mammals—Development of two diagnostic approaches. Ann N Y Acad Sci. 2002; 969 (1): 51–9. 12381563 10.1111/j.1749-6632.2002.tb04350.x

[33] Resendes AR, Almeria S, Dubey JP, Obon E, Juan-Salles C, Degollada E, et al Disseminated toxoplasmosis in a Mediterranean pregnant Risso's dolphin (*Grampus griseus*) with transplacental fetal infection. J Parasitol. 2002; 88 (5): 1029–32. doi: 10.1645/0022-3395(2002)088[1029:DTIAMP]2.0.CO;2 12435153 10.1645/0022-3395(2002)088[1029:DTIAMP]2.0.CO;2

[34] Gonzales-Viera O, Marigo J, Ruoppolo V, Rosas FCW, Kanamura CT, Takakura C, et al Toxoplasmosis in a Guiana dolphin (*Sotalia guianensis*) from Parana, Brazil. Vet Parasitol. 2012; 191 (3–4): 358–62. doi: 10.1016/j.vetpar.2012.09.012 23063774 10.1016/j.vetpar.2012.09.012

[35] Buck JD, Overstrom NA, Patton GW, Anderson HF, Gorzelany JF. Bacteria associated with stranded cetaceans from the northeast USA and southwest Florida Gulf coasts. Dis Aquat Organ. 1991; 10 (2): 147–52.

[36] Stone JR. Diseases of small and medium-sized blood vessels In: Buja LM, Butany J, editors. Cardiovascular Pathology. 4th ed Oxford, UK: Academic Press, Elsevier; 2016 pp. 125–68.

[37] Baker CS, Herman LM. Aggressive behavior between humpback whales (*Megaptera novaeangliae*) wintering in Hawaiian waters. Can J Zool. 1984; 62 (10): 1922–37.

[38] Moore MJ, Der Hoop J, Barco SG, Costidis AM, Gulland FM, Jepson PD, et al Criteria and case definitions for serious injury and death of pinnipeds and cetaceans caused by anthropogenic trauma. Dis Aquat Organ. 2013; 103 (3): 229–64. doi: 10.3354/dao02566 23574708 10.3354/dao02566

[39] Jones EC. *Isistius brasiliensis*, a squaloid shark, theprobable cause of crater wounds on fishes and cetaceans. Fish Bull. 1971; 69 (4): 791–8.

[40] Shirai S, Nakaya K. Functional morphology of feeding apparatus of the Cookiecutter Shark, *Isistius brasiliensis*, (Elasmobranchii, Dalatiinae). Zool Sci 1992; 9: 811–21.

[41] Bornatowski H, Wedekin LL, Heithaus MR, Marcondes MCC, Rossi-Santos MR. Shark scavenging and predation on cetaceans at Abrolhos Bank, eastern Brazil. J Mar Biol Assoc U K. 2012; 92 (8): 1767–72.

[42] Lowry D, C A.L.F., Mara K, Whitenack LB, Delius B, Burgess GH, et al Determining shark size from forensic analysis of bite damage. ‎ Mar Biol. 2009; 156: 2483–92.

[43] Arbelo M, Los Monteros AE, Herraez P, Andrada M, Sierra E, Rodriguez F, et al Pathology and causes of death of stranded cetaceans in the Canary Islands (1999–2005). Dis Aquat Organ. 2013; 103 (2): 87–99. doi: 10.3354/dao02558 23548359 10.3354/dao02558

[44] Lair S, Measures LN, Martineau D. Pathologic findings and trends in mortality in the beluga (*Delphinapterus leucas*) population of the St. Lawrence Estuary, Quebec, Canada, from 1983 to 2012. Vet Pathol. 2016; 53 (1): 22–36. doi: 10.1177/0300985815604726 26374277 10.1177/0300985815604726

[45] Díaz-Delgado J. Pathology and causes of death in stranded cetaceans in the Canary Islands (2006–2012). Doctoral thesis. University of Las Palmas of Gran Canaria.2015 Available from: http://hdl.handle.net/10553/17258.

[46] Gooding CA, Gregory GA, Taber P, Wright RR. An experimental model for the study of meconium aspiration of the newborn. Radiology. 1971; 100 (1): 137–40. doi: 10.1148/100.1.137 5147021 10.1148/100.1.137

[47] Caswell JL, Williams KJ. Respiratory system In: Maxie MG, editor. Jubb, Kennedy and Palmer's Pathology of Domestic Animals. 6th ed Missouri, US: Elsevier; 2016 pp. 465–591.

[48] Matthay MA, Zimmerman GA. Acute lung injury and the acute respiratory distress syndrome: four decades of inquiry into pathogenesis and rational management. Am J Respir Cell Mol Biol. 2005; 33 (4): 319–27. doi: 10.1165/rcmb.F305 16172252 10.1165/rcmb.F305 PMC2715340

[49] Wheeler AP, Bernard GR. Acute lung injury and the acute respiratory distress syndrome: a clinical review. Lancet. 2007; 369 (9572): 1553–64. doi: 10.1016/S0140-6736(07)60604-7 17482987 10.1016/S0140-6736(07)60604-7

[50] Jauniaux T, Petitjean D, Brenez C, Borrens M, Brosens L, Haelters J, et al Post-mortem findings and causes of death of harbour porpoises (*Phocoena phocoena*) stranded from 1990 to 2000 along the coastlines of Belgium and Northern France. J Comp Pathol. 2002; 126 (4): 243–53. doi: 10.1053/jcpa.2001.0547 12056772 10.1053/jcpa.2001.0547

[51] Tanaka M, Izawa T, Kuwamura M, Ozaki M, Nakao T, Ito S, et al A case of meconium aspiration syndrome in a bottlenose dolphin (*Tursiops truncatus*) calf. J Vet Med Sci. 2014; 76 (1): 81–4. doi: 10.1292/jvms.13-0227 23966011 10.1292/jvms.13-0227 PMC3979938

[52] Joseph BE, Duffield DA, Robeck TR. Summary data on reprodution of bottlenose dolphins in controlled environments In: Duffield D, Robeck TR, editors. The Bottlenose Dolphin Breeding Workshop. Silver Springs, MD: AZA Marine Mammal Taxon Advisory Group; 2000 pp. 43–56.

[53] Maitra A. Diseases of infancy and childhood In: Kumar V, Abbas AK, Aster JC, editors. Robbins and Cotran pathologic basis of disease. 9th ed Philadelphia, PA: Elsevier; 2015 pp. 451–82.

[54] Tomilin AG. Mammals of the U.S.S.R. and adjacent countries, Vol. 9, Cetacea. Izdatel'stvo Akademiya Nauk SSSR, Moscow 1957 (Series begun by S I Ognev) (Translated from Russian by Israel Program for Scientific Translantions) xxi + 717 pgs 1967. 1967: 739.

[55] Engel MH. Comportamento reprodutivo da baleia jubarte (*Megaptera novaeangliae*) em Abrolhos Anais de Etologia.; 1996 16–19 de outubro; Uberlândia, MG Sociedade Brasileira de Etologia pp. 275–84.

[56] Deaville R, Jepson PD. UK Cetacean Strandings Investigation Programme, final report for the period 1st January 2005 – 31st December 2010. Department for Environment, Food and Rural Affairs. 2011. 98 p.

[57] Holyoake C, Stephens N, Coughran D. Collection of baseline data on humpback whale (*Megaptera novaeangliae*) health and causes of mortality for long-term monitoring in Western Australia. Australia: Report Department of Conservation and Environment. 2011. 157 p.

[58] Braithwaite JE, Meeuwig JJ, Letessier TB, Jenner KCS, Brierley AS. From sea ice to blubber: linking whale condition to krill abundance using historical whaling records. Polar Biol. 2015; 38: 1195–202.

[59] Lund T, Onarheim H, Reed RK. Pathogenesis of edema formation in burn injuries. World J Surg. 1992; 16 (1): 2–9. 1290261 10.1007/BF02067107

[60] Smith RW, O'neill TJ, Hammond JE. Burn injury: sunlight and a single dose of methoxsalen. Burns Incl Therm Inj. 1984; 10 (6): 420–1. 6478288 10.1016/0305-4179(84)90082-2

[61] Braye F, Latarjet J, Foyatier JL, Comparin JP, Tranchand P, Boucaud C. Extensive burns caused by the abusive use of photosensitizing agents. J Burn Care Rehabil. 1997; 18 (4): 321–5. 9261698 10.1097/00004630-199707000-00008

[62] Gilchrest BA, Soter NA, Stoff JS, Mihm MC Jr. The human sunburn reaction: histologic and biochemical studies. J Am Acad Dermatol. 1981; 5 (4): 411–22. 7287956 10.1016/s0190-9622(81)70103-8

[63] Kumar V, Abbas AK, Aster JC. Environmental and nutritional diseases In: Kumar V, Abbas AK, Aster JC, editors. Robbins and Cotran pathologic basis of disease. 9th ed Philadelphia, PA: Elsevier; 2015 pp. 403–50.

[64] Cowan DF, Curry BE. Histopathology of the alarm reaction in small odontocetes. J Comp Pathol. 2008; 139 (1): 24–33. doi: 10.1016/j.jcpa.2007.11.009 18555267 10.1016/j.jcpa.2007.11.009

[65] Herr H, Cho HJ, Yu S. Burns caused by accidental overdose of photochemotherapy (PUVA). Burns. 2007; 33 (3): 372–5. doi: 10.1016/j.burns.2006.07.005 17218059 10.1016/j.burns.2006.07.005

[66] Rowntree VJ. Feeding, distribution, and reproductive behavior of cyamids (Crustacea: Amphipoda) living on humpback and right whales. Can J Zool. 1996; 74 (1): 103–9.

[67] Paterson RA, Quayle CJ, Dyck SM. A humpback whale calf and two subadult dense-beaked whales recently stranded in southern Queenland. Mem Queensl Mus. 1993; 33 (1): 291–7.

[68] Paterson RA, Dyck SV. Studies of two humpback whales, *Megaptera novaeangliae*, stranded at Fraser Island, Queensland. Mem Queensl Mus. 1991; 30 (2): 343–50.

[69] Herraez P, Espinosa De Los Monteros A, Fernandez A, Edwards JF, Sacchini S, Sierra E. Capture myopathy in live-stranded cetaceans. Vet J. 2013; 196 (2): 181–8. doi: 10.1016/j.tvjl.2012.09.021 23146174 10.1016/j.tvjl.2012.09.021

[70] Jaber JR, Perez J, Arbelo M, Andrada M, Hidalgo M, Gomez-Villamandos JC, et al Hepatic lesions in cetaceans stranded in the Canary Islands. Vet Pathol. 2004; 41 (2): 147–53. doi: 10.1354/vp.41-2-147 15017028 10.1354/vp.41-2-147

[71] Cowan DF, Smith TL. Morphology of the lymphoid organs of the bottlenose dolphin, *Tursiops truncatus*. J Anat. 1999; 194 (4): 505–17. 10445819 10.1046/j.1469-7580.1999.19440505.x PMC1467950

[72] Jauniaux T, Charlier G, Desmecht M, Haelters J, Jacques T, Losson B, et al Pathological findings in two fin whales (*Balaenoptera physalus*) with evidence of morbillivirus infection. J Comp Pathol. 2000; 123 (2–3): 198–201. doi: 10.1053/jcpa.2000.0395 11032676 10.1053/jcpa.2000.0395

[73] Mazzariol S, Marcer F, Mignone W, Serracca L, Goria M, Marsili L, et al Dolphin Morbillivirus and *Toxoplasma gondii* coinfection in a Mediterranean fin whale (*Balaenoptera physalus*). BMC Vet Res. 2012; 8: 20 doi: 10.1186/1746-6148-8-20 22397492 10.1186/1746-6148-8-20 PMC3319419

[74] Rowles TK, Schwacke LS, Wells RS, Saliki JT, Hansen L, Hohn A, et al Evidence of susceptibility to morbillivirus infection in cetaceans from the United States. Mar Mammal Sci. 2011; 27 (1): 1–19.

[75] Jacob JM, West KL, Levine G, Sanchez S, Jensen BA. Initial characterization of novel beaked whale morbillivirus in Hawaiian cetaceans. Dis Aquat Organ. 2016; 117 (3): 215–27. doi: 10.3354/dao02941 26758655 10.3354/dao02941

