# Supplementary figures and images for: Pathology and causes of death in stranded humpback whales (Megaptera novaeangliae) from Brazil
Source: PLoS One. 2018 May 16;13(5):e0194872. doi: 10.1371/journal.pone.0194872 (PMC5955494; doi:10.1371/journal.pone.0194872)

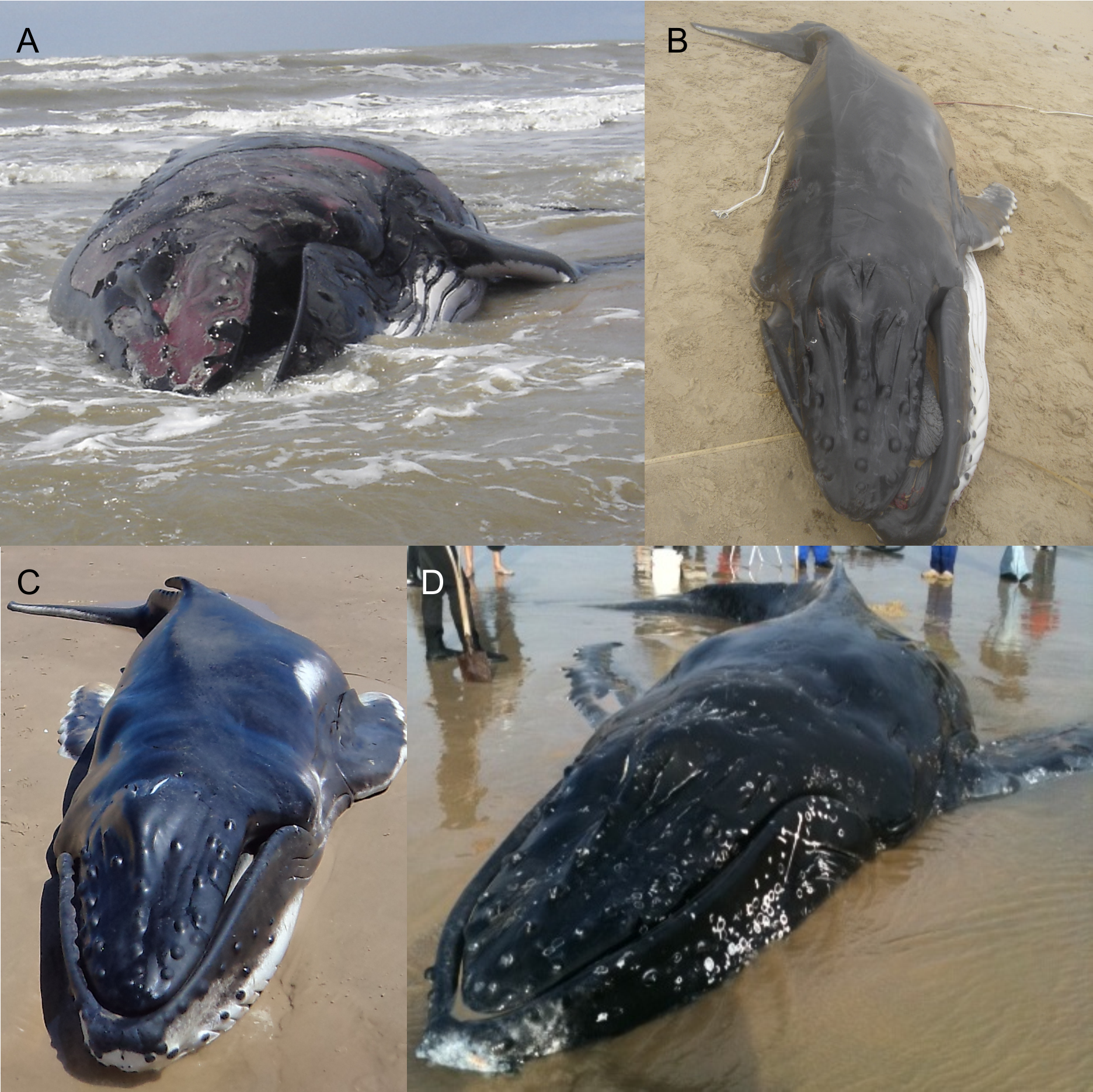

Supplement: S1 Fig — A) Good NS, juvenile (animal no. 3). Epaxial musculature development is appropriate for the age class and fat depots are abundant (not visible), giving a convex dorsal profile. Additionally, this animal has disseminated cutaneous blisters and severe extensive ulcerated areas (sunlight-induced thermal burn; picture taken immediately after euthanasia). B) Moderate NS, calf (animal no. 7). Epaxial muscle development and fat deposits are regarded as normal for the age class, giving a slightly convex or rather straight dorsal profile. C) Poor NS, calf (animal no. 17). Epaxial muscle is mildly decreased and fat depots are reduced (not visible), giving a mild concave dorsal profile. Furthermore, costal and nuchal bony protuberances are slightly prominent. D) Emaciated NS, juvenile (animal no. 15). Epaxial muscle is markedly reduced giving a profound concave dorsal profile. Additionally, costal, nucal and transverse apophyses are prominent and fat depots are markedly depleted or unapparent (not visible). (TIF) [file pone.0194872.s001.tif]

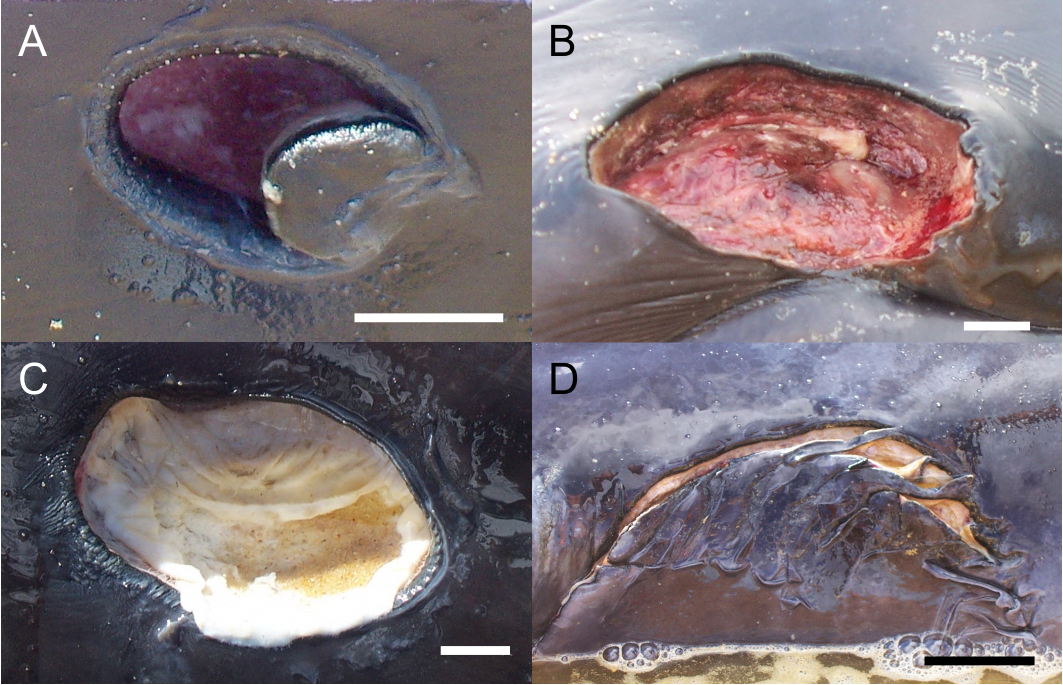

Supplement: S2 Fig — All pictures were taken while the animal was still alive. A) and B) Cutaneous coockiecutter shark (Isistius spp.) with exposure of hyperemic and inflamed dermis and blubber. Bar: 1 cm. C) Cutaneous coockiecutter shark bite with exposed dermis and blubber. This picture is of particular interest because had not this animal being alive, this bite would likely be conservatively considered inflicted post mortem due to insufficient evidences (i.e., hyperemia, hemorrhage, edema, inflammatory exudate). Hence, antemortem bites are likely to be underdiagnosed in regular cetacean autopsies. Bar: 1 cm. D) Cutaneous tiger shark (Galeocerdo cuvier) bite with exposed deep dermis. Similarly to S2C Fig, unequivocal evidences of antemortem wounding are lacking in this bite. Bar: 5 cm. (TIF) [file pone.0194872.s002.tif]

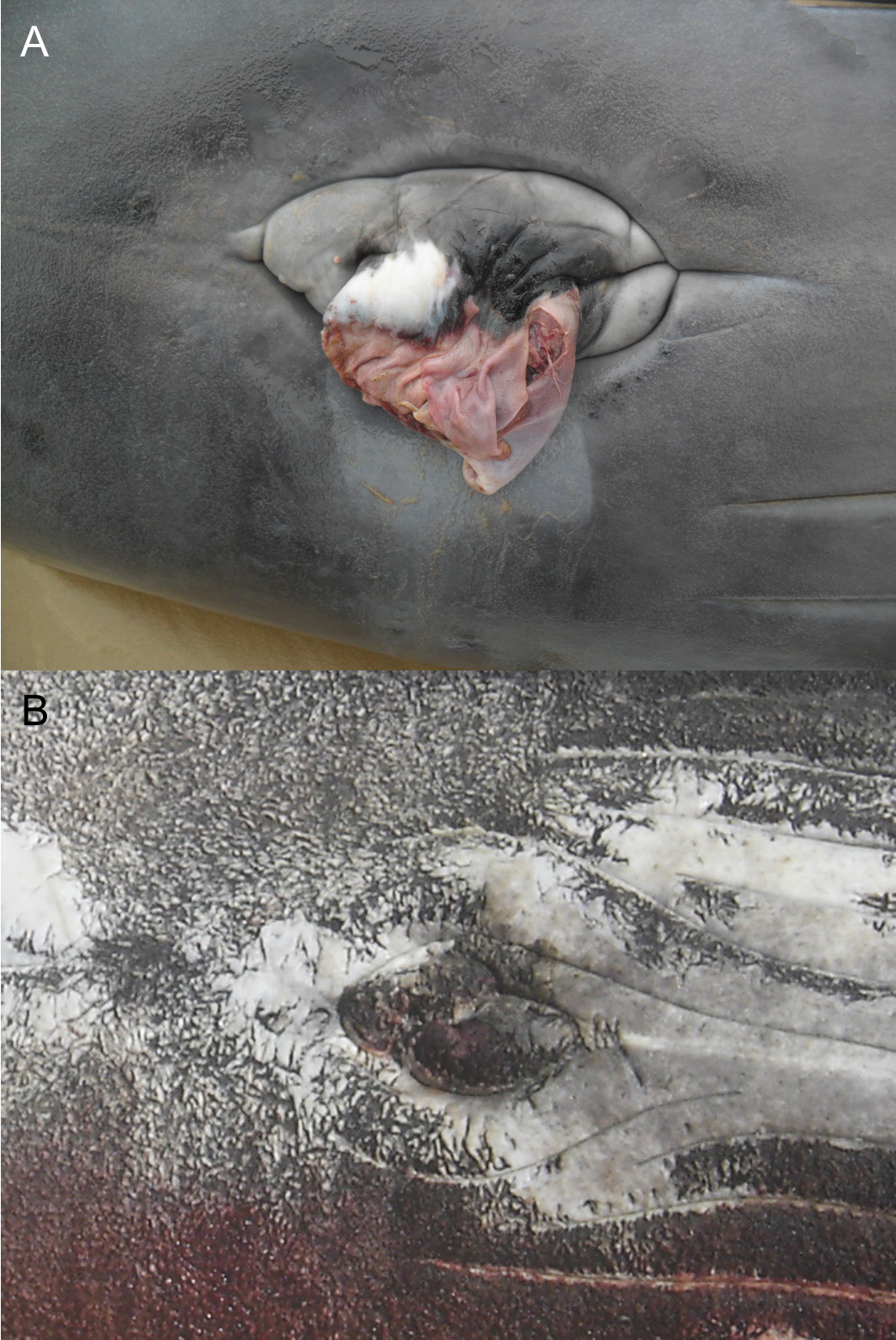

Supplement: S3 Fig — For both images, cranial direction is to the right. A) Umbilical cord (animal no. 12). Presence of irregular, pedunculated and reddened remnants of the umbilical cord. B) Umbilical cord (animal no. 7). The umbilical cord is completely and homogeneously healed. (TIF) [file pone.0194872.s003.tif]
