# Supplementary material for: Pathology and causes of death in stranded humpback whales (Megaptera novaeangliae) from Brazil
Source: PLoS One. 2018 May 16;13(5):e0194872. doi: 10.1371/journal.pone.0194872 (PMC5955494; doi:10.1371/journal.pone.0194872)
Supplement: S1 Table — (DOCX) [file pone.0194872.s004.docx]

**S1 Table**. **Organs tested via immunohistochemistry for morbillivirus and *Toxoplasma gondii* antigens.**

| **Animal number** | ***Morbillivirus*** | ***Toxoplasma gondii*** | |
| --- | --- | --- | --- |
| 1 | Lung, lymph node, thymus | Lung, heart | |
| 2 | Lung, spleen, urinary bladder | Lung, heart, adrenal gland | |
| 3 | Lung, spleen, urinary bladder | Lung, heart, liver | |
| 4 | Lung, cerebrum, cerebellum, urinary bladder | Lung, cerebrum, cerebellum, heart, liver | |
| 5 | Lung | Lung, heart, liver | |
| 6 | Lung, spinal cord | Lung, heart | |
| 7 | Lung, spleen | Heart, liver, adrenal gland | |
| 8 | Lung | Lung, heart, liver | |
| 9 | Spleen | | Spleen, skeletal muscle |
| 10 | Lung, urinary bladder | Lung, heart, liver, skeletal muscle | |
| 11 | NE | NE | |
| 12 | Lung, thymus | Lung, heart, liver, adrenal gland | |
| 13 | Lung | Lung | |
| 14 | Lung, spleen, lymph node | Lung, heart, liver, adrenal gland | |
| 15 | NE | NE | |
| 16 | Lung, spinal cord, cerebrum, cerebellum, lymph node | Lung, cerebrum, cerebellum, heart, liver, skeletal muscle | |
| 17 | Lung, thymus | Lung, heart, liver, skeletal muscle | |
| 18 | Lung, cerebrum, lymph node | Lung, cerebrum, liver | |
| 19 | Lung, spinal cord, cerebrum, urinary bladder | Lung, cerebrum, heart, liver | |
| 20 | Lung, spinal cord, cerebrum | Lung, cerebrum, heart | |
| 21 | Lung, cerebrum, cerebellum | Lung, cerebrum, cerebellum, liver | |
| 22 | NE | NE | |
| 23 | NE | NE | |
| 24 | NE | NE | |

NE: not evaluated.
