# Supplementary material for: Pathology and causes of death in stranded humpback whales (Megaptera novaeangliae) from Brazil
Source: PLoS One. 2018 May 16;13(5):e0194872. doi: 10.1371/journal.pone.0194872 (PMC5955494; doi:10.1371/journal.pone.0194872)
Supplement: S2 Table — (DOCX) [file pone.0194872.s005.docx]

**S2 Table**. **Individual epidemiologic stranding data and signalment of humpback whales stranded in Brazil (2004 – 2016).**

| Animal number | Stranding date | Location | Federal Unit | Sex | BL (m) | Age class | Stranding condition | Nutritional status | Blubber depth (cm) | | | Decomposition code |
| --- | --- | --- | --- | --- | --- | --- | --- | --- | --- | --- | --- | --- |
|  |  |  |  |  |  |  |  |  | **D** | **L** | **V** |  |
| 1 | 07-Oct-2005 | Caravelas | BA | ND | 4.14 | Calf | Dead | ND | NA | NA | NA | MA |
| 2 | 17-Sep-06 | Aracaju | SE | M | 4.06 | Calf | Alive | Moderate | NA | NA | NA | MA |
| 3 | 04-Aug-07 | Belmonte | BA | M | 10.1 | Juvenile | Alive (E) | Good | 11 | 13.5 | 10.5 | MA |
| 4 | 21-Aug-07 | São Mateus | ES | M | 4.37 | Calf | Dead | Good | 3 | 4 | 5 | MA |
| 5 | 08-Sep-07 | Pirambu | SE | F | 4.87 | Calf | Alive | Moderate | NA | NA | NA | MA |
| 6 | 04-Jul-08 | Alcobaça | BA | M | 3.83 | Calf | Alive | Good | 3.5 | 4.5 | 6 | MA |
| 7 | 14-Aug-08 | Conceição da Barra | ES | M | 3.9 | Calf | Alive | Moderate | 3.5 | 3.6 | 3.4 | MA |
| 8 | 04-Jul-09 | Guriri | ES | F | 9.18 | Juvenile | Alive | Good | 19 | 12.5 | 12.5 | MA |
| 9 | 21-Aug-09 | Bitupita | CE | F | 10 | Juvenile | Alive | Good | 14.5 | NA | 16 | Fr |
| 10 | 08-Sep-09 | São Mateus | ES | F | 4.0 | Calf | Alive (E) | Moderate | 3.5 | 6 | 4.5 | Fr |
| 11 | 11-Sep-09 | São Mateus | ES | F | 4.4 | Calf | Dead | Good | NA | NA | NA | AA |
| 12 | 19-Aug-10 | Itaporanga | SE | F | 4.63 | Calf | Dead | Good | NA | NA | NA | Fr |
| 13 | 22-Aug-10 | Capão da Canoa | RS | M | 12.5 | Adult | Alive | Poor | NA | NA | NA | MA |
| 14 | 04-Sep-10 | São Gonçalo do Amarante | CE | M | 3.94 | Calf | Alive | Good | 4.5 | 4 | 5 | Fr |
| 15 | 14-Jul-11 | Balneário Pinhal | RS | F | 7.73 | Juvenile | Alive (E) | Emaciated | NA | NA | NA | MA |
| 16 | 10-Sep-11 | Linhares | ES | M | 3.27 | Calf | Alive | Good | 3.5 | 3 | 3.5 | MA |
| 17 | 06-Sep-12 | Aracaju | SE | F | 4.5 | Calf | Dead | Poor | 5 | 3 | 4 | Fr |
| 18 | 08-Sep-13 | Barra do Riacho | ES | M | 3.5 | Calf | Alive | Good | 4 | 3 | 5.5 | MA |
| 19 | 30-Sep-13 | Prado | BA | M | 4.0 | Calf | Alive | Good | 3.5 | 3.6 | 4.5 | MA |
| 20 | 10-Oct-13 | Santa Cruz de Cabrália | BA | M | 4.5 | Calf | Alive | Good | 5 | 3 | 4 | MA |
| 21 | 09-Oct-14 | Alcobaça | BA | F | 5.15 | Calf | Alive | Good | 6 | 4.5 | 5 | MA |
| 22 | 31-Jul-16 | Porto Seguro | BA | M | 3.74 | Calf | Alive | Moderate | 3.8 | 4.3 | 4.5 | Fr |
| 23 | 10-Aug-16 | São Mateus | ES | M | 4.27 | Calf | Alive (E) | Poor | 2.5 | 3.2 | 5 | Fr |
| 24 | 30-Aug-16 | Linhares | ES | M | 4.54 | Calf | Alive (E) | Moderate | 4.5 | 4 | 3 | Fr |

BA - Bahia; SE - Sergipe; ES - Espírito Santo; CE - Ceará; RS - Rio Grande do Sul; M - male; F - female; ND - Not determined; BL - body length; (E) - euthanized; D – dorsal; L – lateral; V – ventral; NA – not available; Fr - fresh; MA - moderate autolysis; AA - advanced autolysis.
