# Supplementary material for: Pathology and causes of death in stranded humpback whales (Megaptera novaeangliae) from Brazil
Source: PLoS One. 2018 May 16;13(5):e0194872. doi: 10.1371/journal.pone.0194872 (PMC5955494; doi:10.1371/journal.pone.0194872)
Supplement: S3 Table — (DOCX) [file pone.0194872.s006.docx]

**S3 Table**. **Main gross and histologic findings in calf humpback whales stranded in Brazil (2004 – 2016).**

| **Animal number** | **Gross findings** | | **Histopathologic findings** | | | **Diagnosis** | | |  |
| --- | --- | --- | --- | --- | --- | --- | --- | --- | --- |
| **1** | Pulmonary edema | | Pulmonary edema with numerous intra-alveolar squames and atelectasis intercalated with emphysema. | | | Neonatal respiratory distress | | |  |
| **2** | Pulmonary edema | | Pulmonary edema with numerous intra-alveolar squames and histiocytosis; Multicentric lymphoid depletion; Systemic acute serosal hemorrhage | | | Neonatal respiratory distress | | |  |
| **4** | Pulmonary edema and atelectasis; Multifocal cutaneous cookiecutter shark (*Isistius* sp.) bites around the blowhole | | Pulmonary edema with numerous intra-alveolar squames, hyaline membranes, histiocitosis and atelectasis | | | Neonatal respiratory distress | | |  |
| **5** | Pulmonary edema; Multifocal cutaneous cookiecutter shark and tiger shark (*Galeocerdo cuvier*; Supplemental Figure 2) bites^b^ | | Pulmonary edema with intra-alveolar squames; Systemic acute serosal hemorrhages | | | Neonatal respiratory distress; Trauma (ITEI) | | |  |
| **6** | Pulmonary edema and atelectasis; Multifocal cutaneous cookiecutter shark bites^b^ | | Pulmonary edema with intra-alveolar squames, histiocytosis, aspirated (sea water, sand) material, and atelectasis. | | | Neonatal respiratory distress | | |  |
| **7** | Pulmonary edema and atelectasis. | | Pulmonary edema and atelectasis; Multicentric lymphoid depletion; Systemic acute serosal hemorrhages | | | Neonatal respiratory distress | | |  |
| **10** | | Suppurative bronchopneumonia; Pulmonary edema; Hemothorax; *Escherichia coli* grown in pure culture from blowhole swab, and abundant colonies of *E. coli* and *Aeromonas hydrophila* were isolated from lung tissue^b^ | | | Suppurative bronchopneumonia with intralesional bacilli, occasional intra-alveolar squames, edema and atelectasis; Multifocal, acute hemorrhagic gastroenteritis; Multifocal acute tubular degeneration and necrosis; Multicentric lymphoid depletion; Acute hepatocellular degeneration and necrosis with mild dissociation and scattered hemorrhage; Gram-negative bacillary bacterial colonies in alveolar spaces and in blood vessels in the lung, heart and intestine | | | Septicemia associated with *E. coli* and *A. hydrophila* infection | |
| **11** | | Presence of three deep parallel cutaneous linear incised wounds, dorso-laterally located just cranial to the dorsal fin; Multifocal subcutaneous hematomas; Focally extensive pulmonary suffusive hemorrhage^b^ | | | NE | | | Boat collision | |
| **12** | | Pulmonary edema; Multifocal subcutaneous hemorrhage and congestion; Multifocal cutaneous superficial excoriations suggestive of net markings^b^ | | | Pulmonary edema with occasional intra-alveolar squames; Multicentric lymphoid depletion; Systemic acute serosal haemorrhage; Extramedullary hematopoiesis (spleen, lung) | | | Neonatal respiratory distress | |
| **14** | | Pulmonary edema^b^ | | | Pulmonary edema with numerous intra-alveolar squames, hyaline membranes and atelectasis; Multifocal, acute enteritis; Systemic acute serosal haemorrhages | | | Neonatal respiratory distress | |
| **16** | | Pulmonary edema; Multifocal cephalic and cervical subcutaneous and epidural and subarachnoid hematomas, hemorrhage and congestion | | Pulmonary edema with intra-alveolar meconium, squames, hyaline membranes, and acute interstitial pneumonia; Focally extensive leptomeningeal and neuroparenchymal hemorrhage | | | Neonatal respiratory distress | |  |
| **17** | | Pulmonary edema; Multifocal cutaneous superficial excoriations suggestive of net markings^b^ | | Pulmonary edema with intra-alveolar squames, hyaline membranes, rare intra-alveolar bacteria and acute interstitial pneumonia; Multicentric lymphoid depletion | | | Neonatal respiratory distress | |  |
| **18** | | Suffusive hemorrhages in the costal musculature associated with two fractured ribs (4^th^ and 5^th^, left side); Hemothorax; Pulmonary edema | | Pulmonary hemorrhage with edema, hyaline membranes, intra-alveolar squames, and mixed bacteria; Marked systemic serosal hemorrhage | | | Trauma (unknown origin); Neonatal respiratory distress | |  |
| **19** | | Multifocal cervical epidural and subarachnoid hemorrhage; Pulmonary hemorrhage and edema; Hemoperitoneum^b^ | | Pulmonary hemorrhage and edema; Multifocal skeletal muscle necrosis; Systemic acute serosal hemorrhage | | | Trauma (unknown origin) | |  |
| **20** | | Pulmonary edema; Mild cutaneous infestation by whale lice (*Cyamus* sp.) | | Pulmonary edema with intra-alveolar squames; Systemic acute serosal haemorrhage | | | Neonatal respiratory distress | |  |
| **21** | | Pulmonary edema; Marked congestion of skeletal muscle and subcutis with hemorrhages in dorso-cervical region; Multifocal cutaneous superficial excoriations suggestive of net markings^b^ | | Pulmonary edema with intra-alveolar squames and focal arterial cartilaginous embolus; Systemic acute hemorrhage | | | Trauma (unknown origin); Neonatal respiratory distress | |  |
| **22** | | Pulmonary edema; Multifocal hematomas on ventral aspect of shoulder and flippers, caudo-dorsal head, and mandible; Hepatic lipidosis; Multifocal cutaneous superficial excoriations | | Pulmonary edema with intra-alveolar keratin squames; Focal adrenal arterial cartilage embolus; Hepatocellular hyaline globules with pink points and strands; Multicentric sinus histiocytosis and erythrocytosis; Systemic congestion with occasional hemorrhage; Mild, diffuse lymphoid nodal and splenic depletion | | | Neonatal respiratory distress | |  |
| **23** | | Fibrinosuppurative omphalitis, umbilical arteritis and urachocystitis; Pulmonary edema and bronchial hemorrhage; Multifocal axial skeletal muscle petechiae and subcutaneous hematomas; Mild hemothorax and hemopericardium; Systemic congestion and thrombosis (lung, spleen); Multifocal cutaneous cookiecutter shark bites; Multifocal cutaneous superficial excoriations suggestive of net markings; Two 1-2 cm in diameter, well-demarcated, pale tan nodules centered on large descending branches of the right coronary artery; Right scapulo-humeral joint luxation with hemarthros;, *Pseudomonas aeruginosa* was isolated from blowhole swab^b^ | | Subacute omphalitis, umbilical arteritis and urachocystitis; Pulmonary edema with intrabronchiolar and intra-alveolar keratin squames, hemorrhage, histiocytosis (with scattered multinucleated giant cells occasionally associated with basophilic extracellular material) and atelectasis; Multifocal thrombosis and interstitial hemorrhage in rete mirabile; Focal splenic thrombosis; Focal ulcerative and neutrophilic dermatitis and glossitis with intralesional gram-negative coccobacilli and thrombosis; Multicentric lymphoid depletion; Systemic congestion with scattered hemorrhage; Congenital coronary artery fibromuscular dysplasia | | | Infectious omphalitis, umbilical arteritis and urachocystitis; Neonatal respiratory distress; Sublethal entanglement | |  |
| **24** | | Omphalitis and urachocystitis; Pulmonary edema and hemorrhage mixed with aspirated sand and water; Mild hemothorax and hemoabdomen; Focal hematoma associated with parallel linear ulcers; Systemic congestion and hemorrhage^b^ | | Fibrinonecrotizing urachocystitis with thrombosis and numerous intralesional gram-negative coccobacilli and omphalitis; Pulmonary edema with scattered keratin squames, histiocytosis and multifocal arterial cartilage emboli; Focal, acute ulcerative and neutrophilic dermatitis with thrombosis and intralesional gram-negative coccobacilli; Multicentric lymphoid depletion with sinus erythrocytosis and erythrophagocytosis; Systemic congestion and hemorrhage; Focal luminal tracheal hemorrhage | | | Bacterial omphalitis and urachocystitis; Neonatal respiratory distress | |  |

NSLO: no significant lesions observed; ND: not determined; NE: not evaluated. ITEI = interspecific interaction. ^a^ This individual had been removed most of the axial skeletal musculature by humans. ^b^ Incomplete umbilical healing
