# Supplementary material for: Pathology and causes of death in stranded humpback whales (Megaptera novaeangliae) from Brazil
Source: PLoS One. 2018 May 16;13(5):e0194872. doi: 10.1371/journal.pone.0194872 (PMC5955494; doi:10.1371/journal.pone.0194872)
Supplement: S4 Table — (DOCX) [file pone.0194872.s007.docx]

**S4 Table**. **Main gross and histologic findings in juvenile and adult humpback whales stranded in Brazil (2004 – 2016).**

| **Animal number** | **Gross findings** | | **Histopathologic findings** | **Diagnosis** | |
| --- | --- | --- | --- | --- | --- |
| **3** | Disseminated epidermal and dermo-epidermal vesicle and bullae formation throughout the dorsal and lateral body surface to diffuse epidermal clefting and loss (ulceration) secondary to prolonged sunlight exposure; Multifocal deep cutaneous ‘tooth rakes’ on the left dorsal surface of the caudal fin (compatible with killer whale, *Orcinus orca*, predation]; Pulmonary edema | | Severe, diffuse dermo-epidermal clefting and superficial dermal necrosis (sunlight-induced thermal burn); Pulmonary edema with haemorrhage; Hepatic lipidosis; Systemic congestion and scattered hemorrhage | Sunlight-induced thermal burn | |
| **8** | Marked cutaneous infestation by whale lice (*Cyamus* sp.) spanning more than 60% of the body surface; Multifocal cutaneous cookiecutter shark bites; Severe discospondylitis affecting two caudal vertebrae; Pulmonary edema | | Pulmonary edema with alveolar histiocytosis; Lymphoplasmacytic interstitial pneumonia with bronchitis; Lymphoplasmacytic pericholangitis | Caudal discospondylitis | |
| **9** | Pulmonary edema | Pulmonary edema; Eosinophilic and lymphoplasmacytic enteritis | | | ND |
| **13** | Marked axial muscle atrophy; Pulmonary edema | Pulmonary edema; Hepatic lipidosis; Lymphoplasmacytic periportal hepatitis; Lymphoplasmacytic bronchitis; Multicentric lymphoid depletion with follicular hyalinosis | | | Emaciation |
| **15** | Marked axial muscle atrophy; Pulmonary edema | Pulmonary edema; Hepatic lipidosis; Lymphoplasmacytic bronchitis; Multicentric lymphoid depletion | | | Emaciation |

NSLO: no significant lesions observed; ND: not determined; NE: not evaluated.
